# A numerical treatment of radiative nanofluid 3D flow containing gyrotactic microorganism with anisotropic slip, binary chemical reaction and activation energy

**DOI:** 10.1038/s41598-017-16943-9

**Published:** 2017-12-05

**Authors:** Dianchen Lu, M. Ramzan, Naeem Ullah, Jae Dong Chung, Umer Farooq

**Affiliations:** 1 Faculty of Science, Jiangsu University, Zhenjiang, Jiangsu China; 2 Department of Computer Science, Bahria University, Islamabad Campus, Islamabad, 44000 Pakistan; 3 Department of Mathematics, Quaid-i-Azam University, Islamabad, Pakistan; 4 Department of Mechanical Engineering, Sejong University, Seoul, 143-747 Korea; 5 Department of Mathematics, COMSATS Institute of Information Technology, Park road, Tarlai Kalan, Islamabad, 45550 Pakistan

## Abstract

A numerical investigation of steady three dimensional nanofluid flow carrying effects of gyrotactic microorganism with anisotropic slip condition along a moving plate near a stagnation point is conducted. Additionally, influences of Arrhenius activation energy, joule heating accompanying binary chemical reaction and viscous dissipation are also taken into account. A system of nonlinear differential equations obtained from boundary layer partial differential equations is found by utilization of apposite transformations. RK fourth and fifth order technique of Maple software is engaged to acquire the solution of the mathematical model governing the presented fluid flow. A Comparison with previously done study is also made and a good agreement is achieved with existing results; hence reliable results are being presented. Evaluations are carried out for involved parameters graphically against velocity, temperature, concentration fields, microorganism distribution, density number, local Nusselt and Sherwood numbers. It is detected that microorganism distribution exhibit diminishing behavior for rising values of bio-convection Lewis and Peclet numbers.

## Introduction

Stagnation flow is because of the impact of fluid on a solid surface. According to Bernoulli’s equation when fluid velocity comes to zero it experiences a maximum pressure. It is because of the fact that kinetic energy of the fluid is converted into pressure that is known as stagnation pressure (static pressure). Due to remarkable applications in engineering and industry, many investigators elaborated the stagnation flow profile under various situations. Amongst these, Hiemenz^1^ initiated 2D flow in the vicinity of stagnation point. Homann^2^ deliberated 2D axisymmetric flow near a stagnation point. This problem is extended by Wang^3^ to a shrinking sheet. Shateyi and Makinde^4^ elaborated the stagnation point flow past a radially stretched surface in attendance of magnetic field and convective boundary conditions. Flow past a stretched cylinder with Soret-Dufour effects in the locality of stagnation point is discussed by Ramzan *et al*.^5^. Flow of a visco-elastic MHD nanofluid with impacts of non-linear thermal radiation is examined by Farooq *et al*.^6^. All the above literature describes the solution for a no-slip condition. However, there are imperative prospects where slip on a rigid surface occur. Amongst these, flow of the rarefied gas was scrutinized by Sharipov and Seleznev^7^. Wang^8^ discussed the partial slip flow over coated or lubricated surface and striated or rough surface. Choi and Kim^9^ inspected the flow over the superhydrophobic surface. Here, in this case no-slip condition is swapped by the partial slip. The flow over a moving plate near a stagnation point in the presence of isotropic slip was analyzed by Wang^10^. In another study, Wang^11^ described the stagnation flow along a stretched surface comprises anisotropic slip. Anisotropy is caused by the surfaces with a stick-slip strip like in superhydrophobic surface, striated roughed surface (applicable in riblet technology for reduction of pressure). See refs^12–15^ for details of and applications of anisotropic slip.

The principal target of constructing nanofluid is to enhance thermal conductivity of the base fluid including ethylene glycol, engine oil and water. This engineered liquid has tremendous applications in high technology and industry. Credit goes to Choi^16^ who developed the concept of nanofluids. Recently, researchers are contributing a lot for the development of nanofluids. Some of the recent investigations regarding nanofluids include study by Sheikholeslami *et al*.^17^ who by utilizing differential transform method discussed time-dependent nanofluid flow. Rashidi *et al*.^18^ found an analytic and numerical solution of viscous water based nanofluid with second order slip condition using fourth order RK method together with shooting iteration method. Dhanai *et al*.^19^ explored multiple solutions of nanofluid mixed convective flow with slip effect past an inclined stretching cylinder. Mehmood *et al*.^20^ using Optimal Homotopy analysis method examined oblique Jeffery nanofluid flow near a stagnation point. Hayat *et al*.^21^ explored analytical solution of Oldroyd-B nanofluid flow with heat generation/absorption past a stretched surface. Some other applications relevant to nanofluid may be found in^22–31^.

The macroscopic convective movement in a fluid by mutual motion of motile microorganisms causes bio-convection^32^. The density of microorganisms is greater than the fluid thus their movement upgrades the density of the fluid. This phenomenon occurs because of the upswing of self-propelled microorganisms which form a stratified layer at the surface. The motion of microorganisms in a liquid is activated by the different stimulators (like phototactic, chemotaxis, gyrotactic etc.). These microorganisms show different responses towards the stimulators, thus creating different bio-convection systems. Comprehensive studies discussing responses of different microorganisms towards external agents (light, magnetic field, oxygen etc.) are disclosed in^33–46^.

The study of nanofluid flow with micro-organisms is a topic that has not yet been explored much and got extensive attention of many researchers because of its enormous applications in different types of micro systems like micro reactors, utilization in various bio-microsystems e.g., enzyme biosensors, in constructing chip-size micro devices to overcome the demerits of nanoparticles, in micro heat cylinder and micro channel heat sinks etc. See^47–51^ for details of the applications of nanofluids with microorganisms. To our knowledge so far no study has been carried out to discuss 3D flow of nanofluid in the vicinity of a stagnation point with gyrotactic microorganisms. In addition to these upshots, impacts of viscous dissipation, binary chemical reaction, Joule heating, non-linear thermal radiation, activation energy and anisotropic slip are also considered.

## Mathematical model

**Figure 1:**
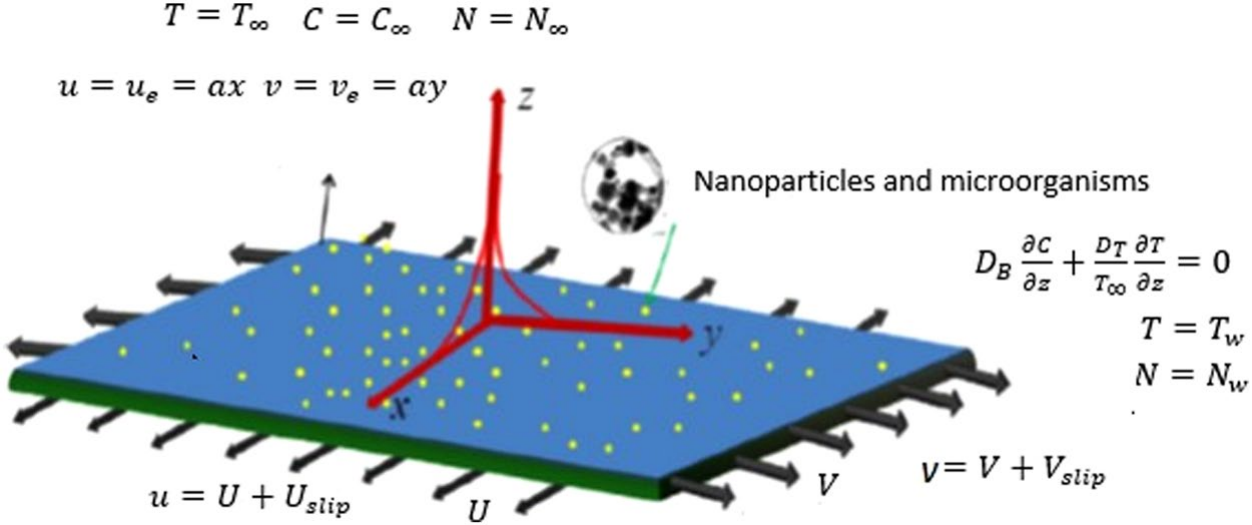
Geometry of the model.

Consider a steady incompressible 3D stagnation point flow of nanofluid on a moving plate. The fluid also contained microorganisms. The flow and heat transfer are inspected under the anisotropic slip, nonlinear thermal radiation effect, viscous dissipation, joule heating, chemical reaction and activation energy effects. The corresponding flow field velocities ($u,\,\,v,\,\,w$) and the frame of reference are adjusted on s uch a pattern that the *x*–axis is with the striations of the plate, the placement of *y*–axis is normal to *x*–axis and the direction of *z*–axis is aligned with the stagnation flow. The motion of plate along *x*– and *y–*axes is uniform with respective velocities (*U*, *V*, O) respectively (See Fig. 1). The flow far from the plate is pressure driven which is considered as

1 $$\begin{array}{c}u=ax,\,v=ay,\,w=-2az,\\ p={p}_{0}-\frac{{a}^{2}{\rho }_{f}}{2}({x}^{2}+{y}^{2})\end{array}$$

Nanofluid suspension is assumed to be stable which is crucial for the existence of microorganisms. Further, to maintain the stability of bioconvection, it is presumed that concentration of the nanoparticles is diluted in water and motion of the microorganisms is free from nanoparticles.

The governing boundary layer equations are given by^52^

2 $$\frac{\partial {u}}{\partial {x}}+\,\frac{\partial {v}}{\partial {y}}+\frac{\partial {w}}{\partial {z}}=0,$$

3 $${u}\frac{\partial {u}}{\partial {x}}+{v}\frac{\partial {u}}{\partial {y}}+{w}\frac{\partial {u}}{\partial {\boldsymbol{z}}}={{a}}^{2}{x}+{{\nu }}_{{\boldsymbol{f}}}\frac{{\partial }^{2}{u}}{\partial {{\boldsymbol{z}}}^{2}},$$

4 $${u}\frac{\partial {v}}{\partial {x}}+{v}\frac{\partial {v}}{\partial {y}}+{w}\frac{\partial {v}}{\partial {\boldsymbol{z}}}={{a}}^{2}{y}+{{\nu }}_{{\boldsymbol{f}}}\frac{{\partial }^{2}{v}}{\partial {{\boldsymbol{z}}}^{2}},$$

5 $$\begin{array}{c}{u}\frac{\partial {T}}{\partial {x}}+{v}\frac{\partial {T}}{\partial {y}}+{w}\frac{\partial {T}}{\partial {\boldsymbol{z}}}=-\frac{1}{{{\rho }}_{{\boldsymbol{f}}}{{c}}_{{\boldsymbol{p}}}}\frac{\partial {{q}}_{{\boldsymbol{r}}}}{\partial {\boldsymbol{z}}}+{\alpha }\frac{{\partial }^{2}{T}}{\partial {{\boldsymbol{z}}}^{2}}+{\tau }\{{{D}}_{{\boldsymbol{B}}}(\frac{\partial {C}}{\partial {\boldsymbol{z}}}\frac{\partial {T}}{\partial {\boldsymbol{z}}})+\frac{{{D}}_{{\boldsymbol{T}}}}{{{T}}_{\infty }}{(\frac{\partial {T}}{\partial {\boldsymbol{z}}})}^{2}\}\\ \quad \quad \quad \quad \quad \quad \quad \quad \,\,\,+\quad \frac{{\mu }}{{{\rho }}_{{\boldsymbol{f}}}{{c}}_{{\boldsymbol{p}}}}\{{(\frac{\partial {u}}{\partial {\boldsymbol{z}}})}^{2}+{(\frac{\partial {v}}{\partial {\boldsymbol{z}}})}^{2}\}+\frac{{\sigma }{{{\beta }}_{0}}^{2}}{{{\rho }}_{{\boldsymbol{f}}}{{c}}_{{\boldsymbol{p}}}}({{u}}^{2}+{{v}}^{2}),\end{array}$$

6 $${u}\frac{\partial {C}}{\partial {x}}+{v}\frac{\partial {C}}{\partial {y}}+{w}\frac{\partial {C}}{\partial {\boldsymbol{z}}}={{D}}_{{\boldsymbol{B}}}\frac{{\partial }^{2}{C}}{\partial {{\boldsymbol{z}}}^{2}}+\frac{{{D}}_{{\boldsymbol{T}}}}{{{T}}_{\infty }}\frac{{\partial }^{2}{T}}{\partial {{\boldsymbol{z}}}^{2}}-{{{K}}_{{\boldsymbol{r}}}}^{2}({C}-{{C}}_{\infty }){(\frac{{T}}{{{T}}_{\infty }})}^{{n}}{{e}}^{-\frac{{{\boldsymbol{E}}}_{{\boldsymbol{a}}}}{{KT}}},$$

7 $${u}\frac{\partial {N}}{\partial {x}}+{v}\frac{\partial {N}}{\partial {y}}+{w}\frac{\partial {N}}{\partial {\boldsymbol{z}}}+\frac{{b}{{W}}_{{\boldsymbol{c}}}}{{\rm{\Delta }}{C}}\frac{\partial }{\partial {\boldsymbol{z}}}({N}\frac{\partial {C}}{\partial {\boldsymbol{z}}})={{D}}_{{\boldsymbol{n}}}\frac{{\partial }^{2}{N}}{\partial {{\boldsymbol{z}}}^{2}}\,,$$

where *u*, *v* and *w* are the velocity components along the *x–*, *y–* and z–axes respectively. *u*_*f*_ is the kinematic viscosity of fluid, *T* is the temperature, *C* is the concentration of nanoparticles, *q*_*r*_ is the radiative heat flux, $\tau =\frac{{({\rho }_{f}c)}_{p}}{{({\rho }_{f}c)}_{f}}$ is the ratio of the effective heat capacity of nanoparticle and base fluid, *α* thermal diffusivity, *D*_*B*_ is Brownian diffusion coefficient, *D*_*T*_ is thermophoretic diffusion coefficient, *β*_0_ is the strength of magnetic field. *N* is concentration of motile microorganisms, *W*_c_ is the maximum cell swimming speed, *b* is the chemotaxis constant and *D*_n_ is the microorganisms diffusion coefficient. The last term on the RHS of equation (6) is the modified Arrhenius equation (see Tencer *et al*.^53^.)

8 $${K}_{r}=B{(\frac{T}{{T}_{\infty }})}^{n}\exp [(\frac{-{E}_{a}}{KT})],$$

with $K=8.61\ast \frac{{10}^{-5}ev}{K}\,$ is the Boltzmann constant, where −1 ≤ *n* ≤ 1 is the fitted rate. Svante Arrhenius first time furnished the idea of the activation energy in 1889 and is considered as the bench mark to estimate the amount of energy needed for reactants to convert into products. Energy in molecules is stockpiled in the form of kinetic or potential energy. This stored energy may be consumed for the performance of a chemical reaction. When the movement of molecules is sluggish with least kinetic energy or break into irregular orientation, chemical reaction is not performed and they simply glance off each other. Nevertheless, if the movement of molecules is enough quick with right collision and alignment to such a level that impact of kinetic energy is more dominating than the base energy bench mark then the chemical reaction takes place. Therefore, the least energy required to perform a chemical reaction is named as activation energy. The notion of activation energy is applicable in many applications like oil emulsions, geothermal and in hydrodynamics.

Pertinent boundary conditions supporting to the present model are

9 $$\begin{array}{c}{u}={U}+{\mu }{{N}}_{1}\frac{\partial {u}}{\partial {\boldsymbol{z}}},\,{v}={V}+{\mu }{{N}}_{2}\frac{\partial {v}}{\partial {\boldsymbol{z}}},\,{w}=0\,,\\ {T}={{T}}_{{\boldsymbol{w}}},\,{{D}}_{{\boldsymbol{B}}}\frac{\partial {C}}{\partial {\boldsymbol{z}}}+\frac{{{D}}_{{\boldsymbol{T}}}}{{{T}}_{\infty }}\frac{\partial {T}}{\partial {\boldsymbol{z}}}=0,\,{N}={{N}}_{{\boldsymbol{w}}}\,{at}\,{\boldsymbol{z}}=0,\\ {u}\to {ax},\,{v}\to {ay},\,{w}\to -2{az},\,{T}\to {{T}}_{\infty ,}\\ {C}\to {{C}}_{\infty ,},\,{N}\to {{N}}_{\infty ,}\,{at}\,{z}\to \infty ,\end{array}$$

where *N*_1_, *N*_2_, *T*_*w*_ and *N*_*w*_ are the slip coefficients along *x*– axis, *y–*axis, temperature and concentration of microorganisms at the surface respectively whereas ${T}_{\infty }$, ${C}_{\infty }$ and ${N}_{\infty }$ are the temperature, concentration distribution of nanoparticle and microorganisms far away from the surface respectively.

Introducing following similarity transformations

10 $$\begin{array}{c}{\eta }=\sqrt{\frac{{a}}{{{\nu }}_{{\boldsymbol{f}}}}{\boldsymbol{z}}},\,{u}={ax}{f}{^{\prime} }({\eta })+{Uh}({\eta }),\,{v}={ay}{g}{^{\prime} }({\eta })+{Vk}({\eta }),\,\\ {w}=\sqrt{-{a}{{\nu }}_{{\boldsymbol{f}}}}({f}({\eta })+{g}({\eta })\,),\,{\theta }({\eta })=\frac{{T}-{{T}}_{\infty }}{{{T}}_{{\boldsymbol{w}}}-{{T}}_{\infty }},\,\varphi ({\eta })=\frac{{C}-{{C}}_{\infty }}{{{C}}_{\infty }},\,{\xi }=\frac{{N}}{{{N}}_{{w}}}.\end{array}$$

Following Rosseland approximation we can write

11 $${{q}}_{{\boldsymbol{r}}}=-\frac{4{{\sigma }}^{\ast }}{3{{k}}^{\ast }}\frac{\partial {{T}}^{4}}{\partial {\boldsymbol{z}}}=\frac{16{{\sigma }}^{\ast }}{3{{k}}^{\ast }}\frac{{{T}}^{3}\partial {T}}{\partial {\boldsymbol{z}}}$$

where σ^*^ denotes Stefan Boltzmann constant and *k* ^*^ is mean absorption coefficient Using the above expression (11), the equation (5) can be simplified as

12 $$\begin{array}{c}u\frac{\partial T}{\partial x}+v\frac{\partial T}{\partial y}+w\frac{\partial T}{\partial z}=\frac{\partial }{\partial z}\{(\alpha +\frac{16{\sigma }^{\ast }{T}^{3}}{3{k}^{\ast }})\frac{\partial T}{\partial z}\}+\alpha \frac{{\partial }^{2}T}{\partial {z}^{2}}+\tau \{{D}_{B}(\frac{\partial C}{\partial z}\frac{\partial T}{\partial z})+\frac{{D}_{T}}{{T}_{\infty }}{(\frac{\partial T}{\partial z})}^{2}\}\\ +\frac{{\mu }}{{{\rho }}_{{\boldsymbol{f}}}{{c}}_{{\boldsymbol{p}}}}\{{(\frac{\partial {u}}{\partial {\boldsymbol{z}}})}^{2}+{(\frac{\partial {v}}{\partial {\boldsymbol{z}}})}^{2}\}+\frac{{\sigma }{{{\beta }}_{0}}^{2}}{{{\rho }}_{{\boldsymbol{f}}}{{c}}_{{\boldsymbol{p}}}}({{u}}^{2}+{{v}}^{2}).\end{array}$$

Using the transformation (10) the first term on the right-hand side of equation (12) can be written as

13 $${\alpha }\frac{\partial }{\partial {\boldsymbol{z}}}\{(1+{Rd}{(1+({Tr}-1){\theta })}^{3})\frac{\partial {T}}{\partial {z}}\}$$

Utilizing (10), equations (3), (4), (6), (7) and (12) with boundary conditions (9) take the form

14 $${f}^{\prime\prime\prime} +({f}+{g}){f}^{\prime\prime} -{{f}^{\prime}2}+1=0,$$

15 $${g}^{\prime\prime\prime} +({f}+{g}){g}^{\prime\prime} -{{g}^{\prime}2}+1=0,$$

16 $${h}^{\prime\prime} +({f}+{g}){h}^{\prime} -{hf}^{\prime} =0,$$

17 $${k}^{\prime\prime} +({f}+{g}){k}{^{\prime} }-{kg}^{\prime} =0,$$

18 $$\begin{array}{c}[\{1+{Rd}{(1+({Tr}-1){\theta })}^{3}\}{\theta }{^{\prime} }]^{\prime} +{\Pr }({f}+{g}){\theta }{^{\prime} }+{\Pr }(\mathrm{Nb}{\theta }{^{\prime} }\varphi ^{\prime} +{Nt}{{\theta }}^{\text{'}2})\\ \quad +{PrEc}({{f}}^{{\prime\prime} 2}+{{g}}^{^{\prime\prime} 2}+{{h}{^{\prime} }}^{2}+{{k}^{{\prime}2}}+2({f}^{\prime\prime} {h}{^{\prime} }+{g}^{\prime\prime} {k}{^{\prime} }))\\ \quad +{PrEcM}({f}{\text{'}}^{2}+{{g}}^{\text{'}2}+{{h}}^{2}+{{k}}^{2}+2({f}{^{\prime} }h+{g}\text{'}{k}))=0,\end{array}$$

19 $$\varphi ^{\prime\prime} +{Sc}({f}+{g})\varphi ^{\prime} +\frac{{Nt}}{{Nb}}{\theta }^{\prime\prime} -{\beta }{Sc}{(1+{\delta }{\theta })}^{{\boldsymbol{n}}}{{e}}^{(\frac{-{\boldsymbol{E}}}{1+{\boldsymbol{\delta }}{\boldsymbol{\theta }}})}=0,$$

20 $${\xi }^{\prime\prime} +{Lb}({f}+{g}){\xi }{^{\prime} }-{Pe}({\xi }{^{\prime} }\varphi ^{\prime} +{\xi }\varphi ^{\prime\prime} )=0,$$

with boundary conditions:

21 $$\begin{array}{c}{f}(0)=0,\,{f}{^{\prime} }(0)={{\lambda }}_{1}{f}^{\prime\prime} (0),\,{g}(0)=0,\,\,{g}{^{\prime} }(0)={{\lambda }}_{2}{g}^{\prime\prime} (0),\\ {h}(0)=1+{{\lambda }}_{1}{h}{^{\prime} }(0),\,{k}(0)=1+{{\lambda }}_{2}{k}{^{\prime} }(0),\,\\ {\theta }(0)=1,\,{Nb}\varphi ^{\prime} (0)+Nt{\theta }{^{\prime} }(0)=0,\,{\xi }(0)=1,\\ {f}{^{\prime} }({\eta })\to 1,\,{g}{^{\prime} }({\eta })\to 1,\,{h}({\eta })\to 0,\,{k}({\eta })\to 0,\,{\theta }({\eta })\to 0,\\ \varphi ({\eta })\to 0,\,{\xi }({\eta })\to 0,as\,{\eta }\to \infty .\end{array}$$

In the above expressions the non dimensional parameters are defined as folows:

$$\begin{array}{c}Rd=\frac{16{\sigma }^{\ast }{T}^{3}}{3{k}^{\ast }{k}_{f}},Tr=\frac{{T}_{w}}{{T}_{\infty }},{P}_{r}=\frac{\alpha }{{\upsilon }_{f}},Ec=\frac{{a}^{2}{x}^{2}}{{c}_{p}{\rm{\Delta }}T},M=\frac{\sigma {{B}_{0}}^{2}}{{\rho }_{f}a}\,,Sc=\frac{{\nu }_{f}}{{D}_{B}},Nt=\frac{\tau {D}_{T}{\rm{\Delta }}T}{{T}_{\infty }{\nu }_{f}},\\ Nb=\frac{\tau {D}_{B}{{\rm{C}}}_{\infty }}{{\nu }_{f}},\beta =\frac{{{K}_{r}}^{2}}{a},\delta =\frac{{\rm{\Delta }}T}{{T}_{\infty }},E=\frac{{E}_{a}}{K{T}_{\infty }},Lb=\frac{{\nu }_{f}}{{D}_{n}},{P}_{e}=\frac{b{W}_{c}}{{D}_{n}},{\lambda }_{i}={N}_{i}\mu \sqrt{\frac{a}{{\nu }_{f}}}\end{array}$$are the radiation parmeter, temperature ratio parameter, Prandtl number, Eckert number, magnetic parameter, Schmidt number, thermophoresis parameter, Brownian motion parameter, chemical reaction parameter, temperature difference parameter, dimensionless activation energy, bioconvection Lewis number, bioconvection, Peclet number and slip parmaters respectively. Nusselt number, a dimensionless parameter, is the quotient of convective to conductive heat transfer perpendicular to the boundary. Likewise, Sherwood number, alternately mass transfer Nusselt number, is a non-dimensional number that describes nanoparticle flux rate in the fluid.

Here, Nusselt numbers $N{u}_{x}$, $N{u}_{y}$ local Sherwood numbers $S{h}_{x}$, $S{h}_{y}$ and local density numbers $N{n}_{x}$, $N{n}_{y}$ along $x$ and $y$ directions are given by

22 $$\begin{array}{c}{N}{{u}}_{{\boldsymbol{x}}}=\frac{{x}{{q}}_{{\boldsymbol{w}}}}{{{k}}_{{\boldsymbol{f}}}{\Delta }T},\,{S}{{h}}_{{\boldsymbol{x}}}=\frac{{x}{{q}}_{{\boldsymbol{m}}}}{{{D}}_{{B}}{\Delta }C},\,{N}{{n}}_{{\boldsymbol{x}}}=\frac{{x}{{q}}_{{\boldsymbol{n}}}}{{{D}}_{{\boldsymbol{n}}}{\Delta }N},\\ {N}{{u}}_{{\boldsymbol{y}}}=\frac{{x}{{q}}_{{\boldsymbol{w}}}}{{{k}}_{{f}}{\Delta }T},\,{S}{{h}}_{{\boldsymbol{y}}}=\frac{{y}{{q}}_{{\boldsymbol{m}}}}{{{D}}_{{B}}{\Delta }C},\,{N}{{n}}_{{\boldsymbol{y}}}=\frac{{y}{{q}}_{{\boldsymbol{n}}}}{{{D}}_{{n}}{\Delta }N},\end{array}$$

where

23 $${{q}}_{{\boldsymbol{w}}}=-{{k}}_{{\boldsymbol{f}}}{(\frac{\partial {T}}{\partial {\boldsymbol{z}}})}_{{\boldsymbol{z}}=0},\,{{q}}_{{\boldsymbol{m}}}=-{{D}}_{{\boldsymbol{B}}}{(\frac{\partial {C}}{\partial {\boldsymbol{z}}})}_{{\boldsymbol{z}}=0},\,{{q}}_{{\boldsymbol{n}}}=-{{D}}_{{\boldsymbol{n}}}{(\frac{\partial {N}}{\partial {\boldsymbol{z}}})}_{{\boldsymbol{z}}=0},$$

Making use of Eq. (10) in Eq. (22) we get

24 $$\begin{array}{c}{R}{{e}}_{{\boldsymbol{x}}}^{-\frac{1}{2}}{N}{{u}}_{{\boldsymbol{x}}}={R}{{e}}_{{\boldsymbol{y}}}^{-\frac{1}{2}}{N}{{u}}_{{\boldsymbol{y}}}=-{\theta }{^{\prime} }(0),\\ {R}{{e}}_{{\boldsymbol{x}}}^{-\frac{1}{2}}{S}{{h}}_{{\boldsymbol{x}}}={R}{{e}}_{{\boldsymbol{y}}}^{-\frac{1}{2}}{S}{{h}}_{{\boldsymbol{y}}}=-\frac{\varphi ^{\prime} (0)}{\varphi (0)},\\ {R}{{e}}_{{\boldsymbol{x}}}^{-\frac{1}{2}}{N}{{n}}_{{\boldsymbol{x}}}={R}{{e}}_{{\boldsymbol{y}}}^{-\frac{1}{2}}{N}{{n}}_{{\boldsymbol{y}}}=-{\xi }{^{\prime} }(0),\end{array}$$

with $R{e}_{x}$ and $R{e}_{y}$ are the Reynolds number along *x*– and *y*– directions.

## Numerical method

A numerical method dsolve command with option numeric built in Maple 18 is utilized in order to solve system of differential equations (14) to (20) along with associated boundary conditions (21). This method uses RK45 technique to interpret the problem *i*.*e*., it corporate both Runge-Kutta fourth and fifth order scheme. The details of the technique are given by

25 $$\begin{array}{c}{{K}}_{0}={f}({{x}}_{{\boldsymbol{n}}},\,{{y}}_{{\boldsymbol{n}}}){h},\\ {{K}}_{1}={f}({{x}}_{{\boldsymbol{n}}}+\frac{1}{4}{h},\,{{y}}_{{\boldsymbol{n}}}+\frac{1}{4}{{K}}_{0}){h},\\ {{K}}_{2}={f}({{x}}_{{\boldsymbol{n}}}+\frac{3}{8}{h},\,{{y}}_{{\boldsymbol{n}}}+\frac{3}{32}{{K}}_{0}+\frac{9}{32}{{K}}_{1}){h},\\ {{K}}_{3}={f}({{x}}_{{\boldsymbol{n}}}+\frac{12}{13}{h},\,{{y}}_{{\boldsymbol{n}}}+\frac{1932}{2197}{{K}}_{0}-\frac{7200}{2197}{{K}}_{1}+\frac{7296}{2197}{{K}}_{2}){h},\\ {{K}}_{4}={f}({{x}}_{{\boldsymbol{n}}}+{h},\,{{y}}_{{\boldsymbol{n}}}+\frac{439}{216}{{K}}_{0}-8{{K}}_{1}+\frac{3680}{513}{{K}}_{2}-\frac{845}{4104}{{K}}_{3}){h},\\ {{K}}_{5}={f}({{x}}_{{\boldsymbol{n}}}+\frac{1}{2}{h},\,{{y}}_{{\boldsymbol{n}}}-\frac{8}{27}{{K}}_{0}+2{{K}}_{1}-\frac{3544}{2565}{{K}}_{2}+\frac{1859}{4104}{{K}}_{3}-\frac{11}{40}{{K}}_{4}){h},\end{array}$$

26 $$\begin{array}{rcl}{{y}}_{{\boldsymbol{n}}+1} & = & \,\,{{y}}_{{\boldsymbol{n}}}+\frac{25}{216}{{K}}_{0}+\frac{1408}{2565}{{K}}_{2}+\frac{2197}{4104}{{K}}_{3}-\frac{1}{5}{{K}}_{4}\\ {{\boldsymbol{z}}}_{{\boldsymbol{n}}+1} & = & {{\boldsymbol{z}}}_{{\boldsymbol{n}}}+\frac{16}{135}{{K}}_{0}+\frac{6656}{12825}{{K}}_{2}+\frac{28561}{56430}{{K}}_{3}-\frac{9}{5}{{K}}_{4}+\frac{2}{55}{{K}}_{5}\end{array}$$

where $y$ and $z$ are fourth and fifth order Runge-Kutta technique, the estimated error can be calculated by subtracting the two obtained values. The new step size can be determined as

27 $${{h}}_{{\boldsymbol{new}}}={{h}}_{{\boldsymbol{old}}}{(\frac{\epsilon {{h}}_{{\boldsymbol{old}}}}{|{{\boldsymbol{z}}}_{{\boldsymbol{n}}+1}-{{y}}_{{\boldsymbol{n}}+1}|})}^{\frac{1}{4}}$$

To approach the asymptotic values given by in equation (21) we have chosen $\,{\eta }_{max}={\eta }_{\infty }=3$.

## Results and Discussions

**Figure 2:**
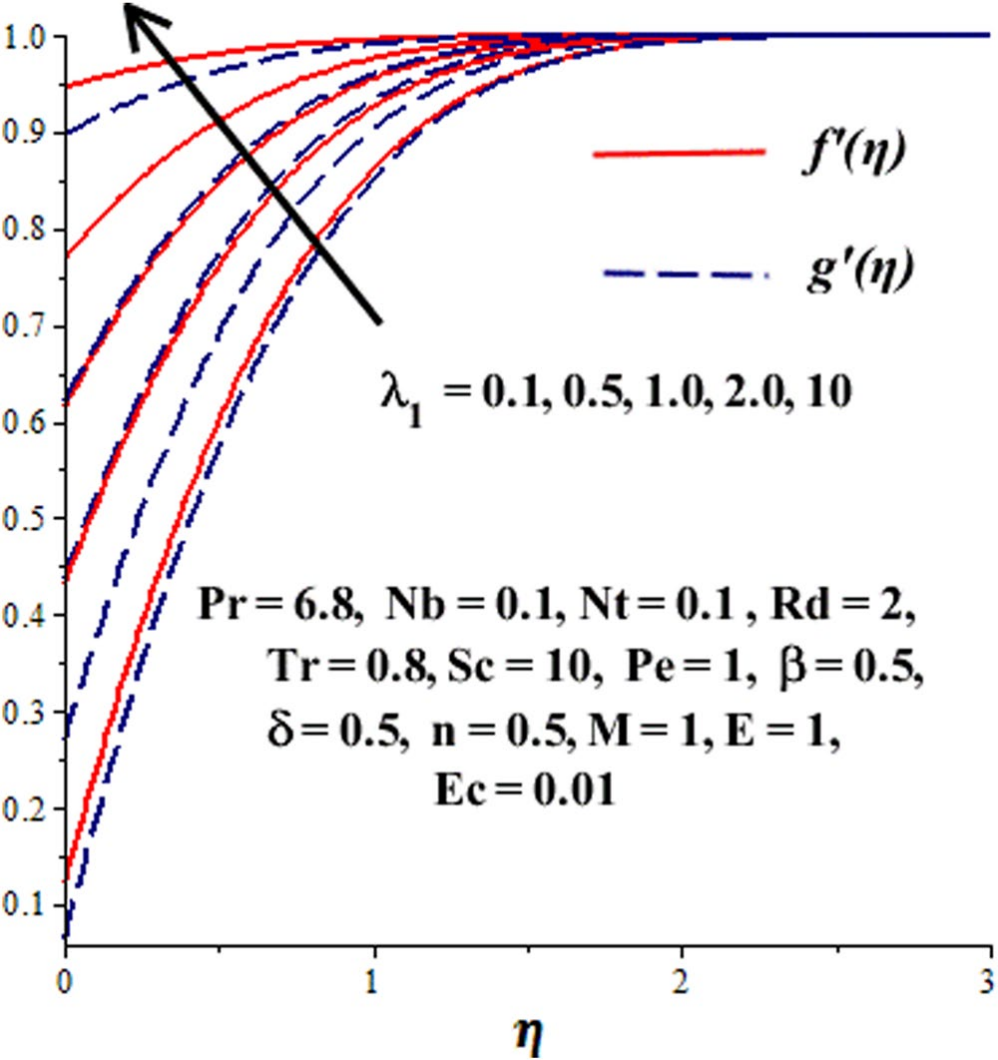
Effect of *λ*_1_ on *f*(*η*), *g*′(*η*) when ${\gamma }=\frac{{{\lambda }}_{2}}{{{\lambda }}_{1}}=0.5$.

**Figure 3:**
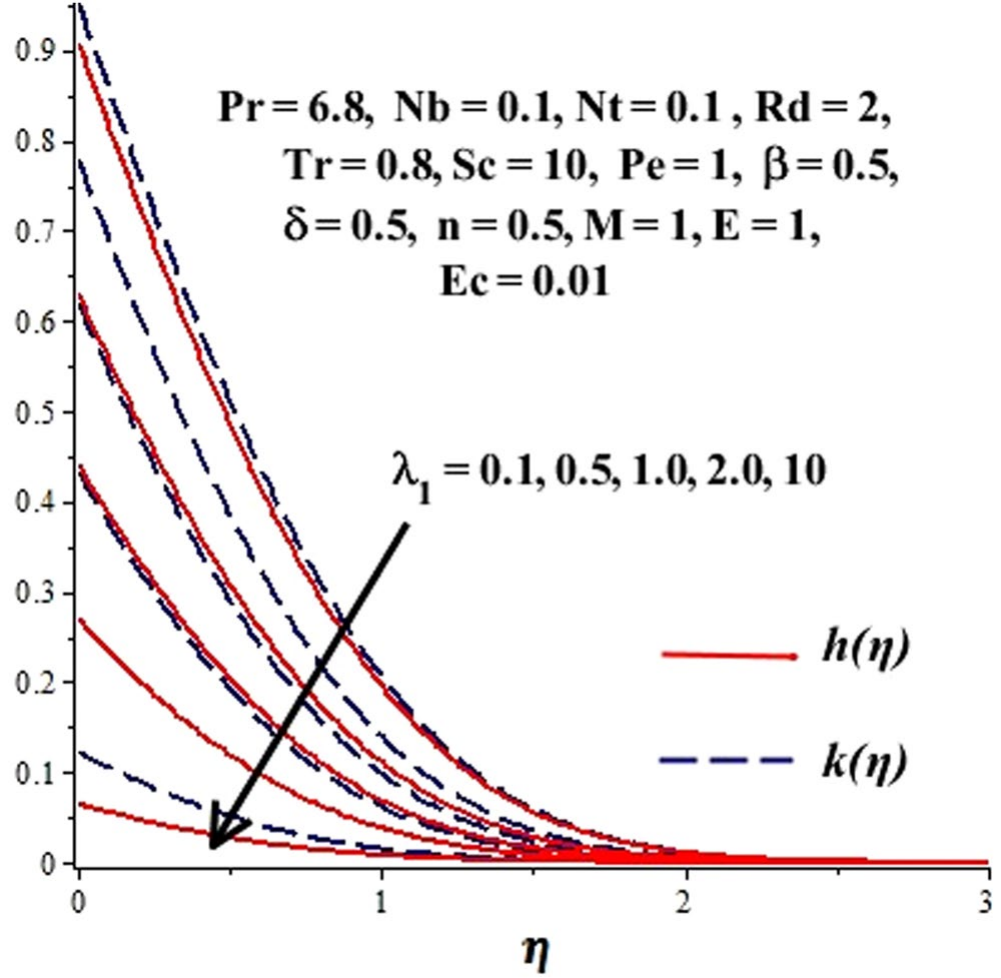
Effect of *λ*_1_ on *h*(*η*), *k*(*η*) when ${\gamma }=\frac{{{\lambda }}_{2}}{{{\lambda }}_{1}}=0.5$.

**Figure 4:**
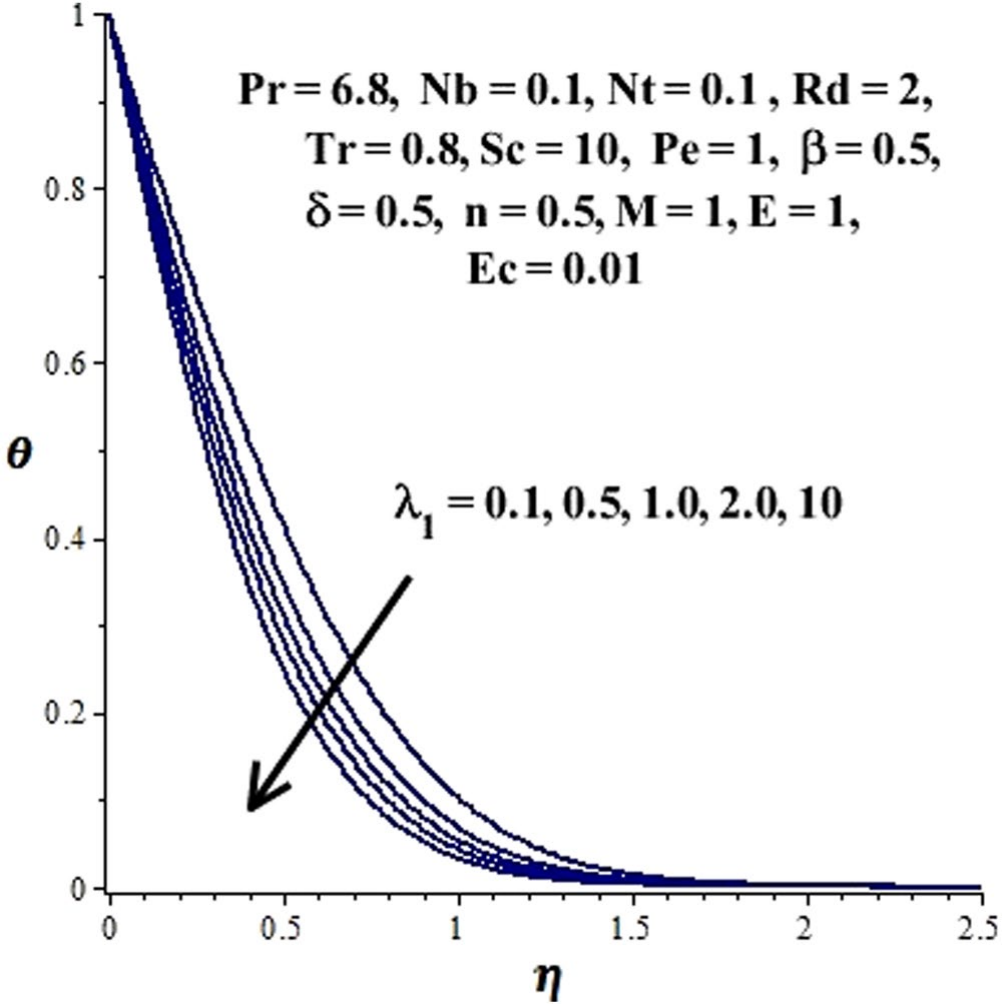
Effect of *λ*_1_ on *θ*(η) when ${\gamma }=\frac{{{\lambda }}_{2}}{{{\lambda }}_{1}}=0.5$.

**Figure 5:**
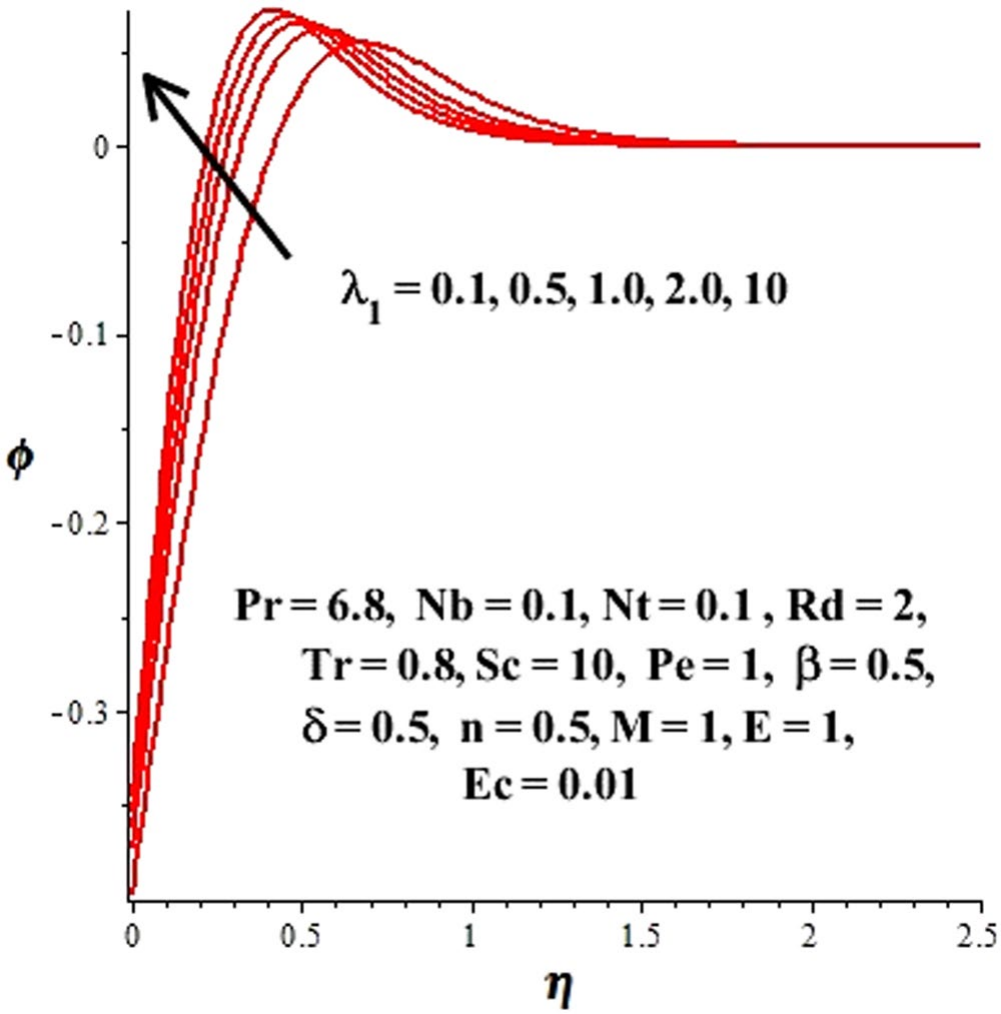
Effect of *λ*_1_ on *ϕ*(*η*) when ${\gamma }=\frac{{{\lambda }}_{2}}{{{\lambda }}_{1}}=0.5$.

**Figure 6:**
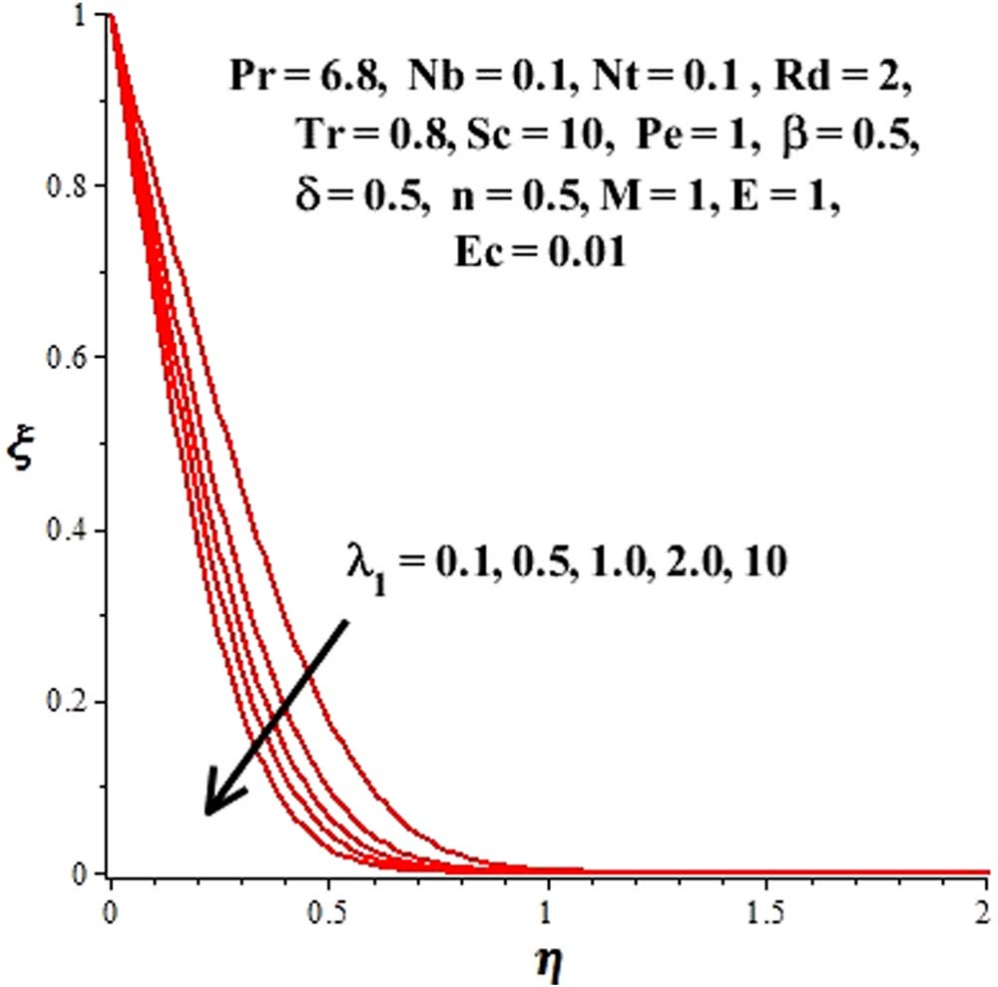
Effect of λ_1_ on ξ(*η*) when ${\gamma }=\frac{{{\lambda }}_{2}}{{{\lambda }}_{1}}=0.5$.

**Figure 7:**
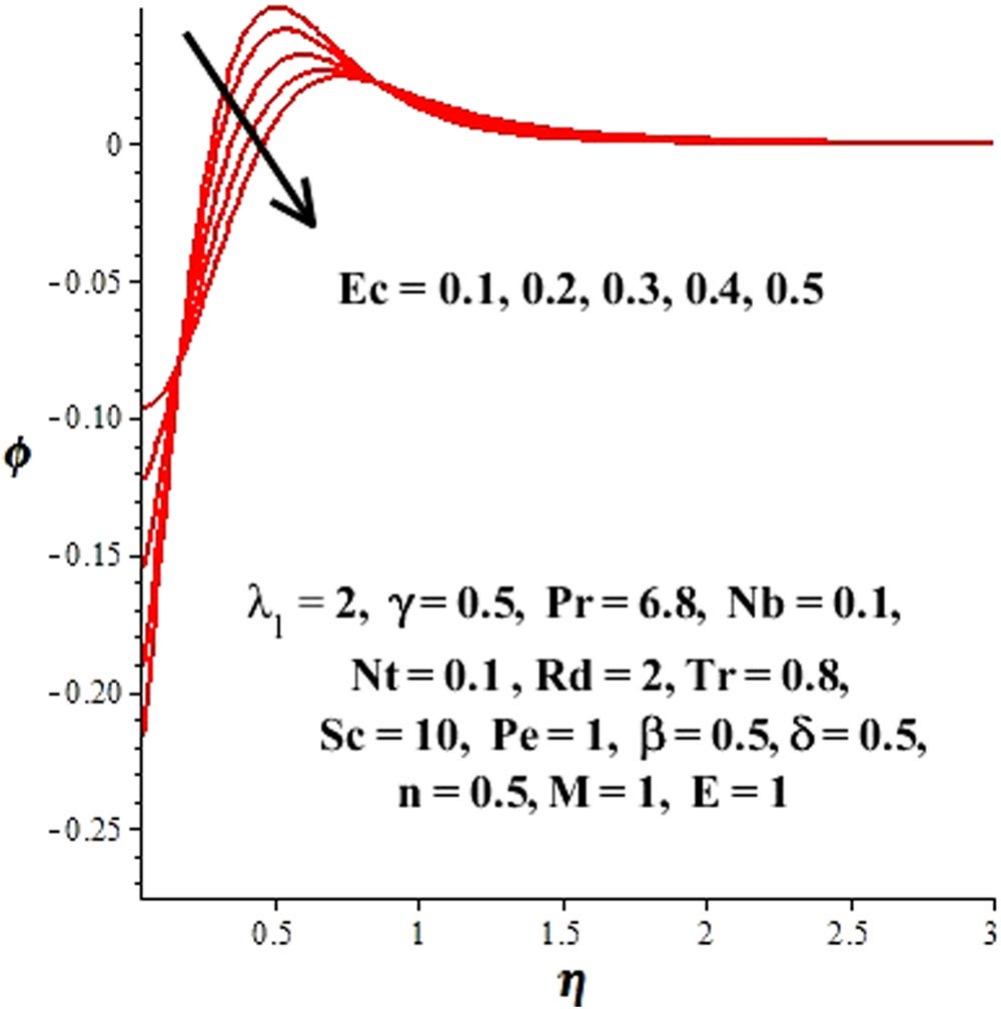
Variation of concentration profile due to Eckert number.

**Figure 8:**
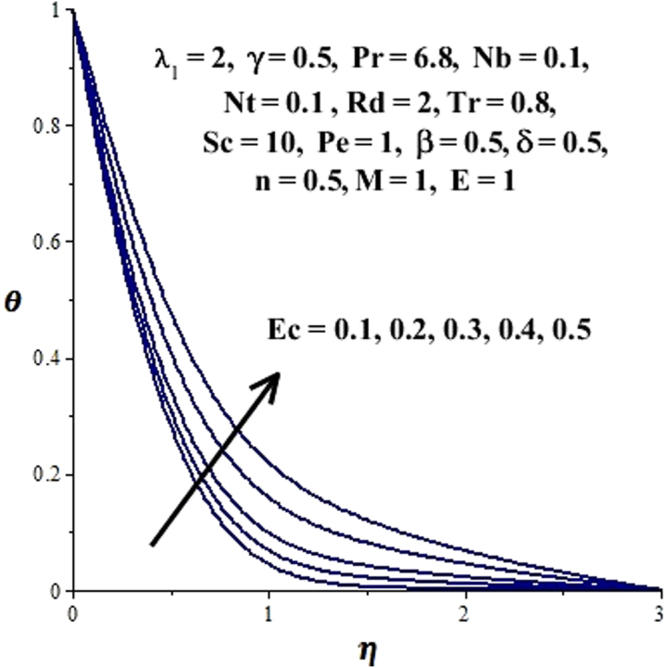
Variation of temperature field due to Eckert number.

**Figure 9:**
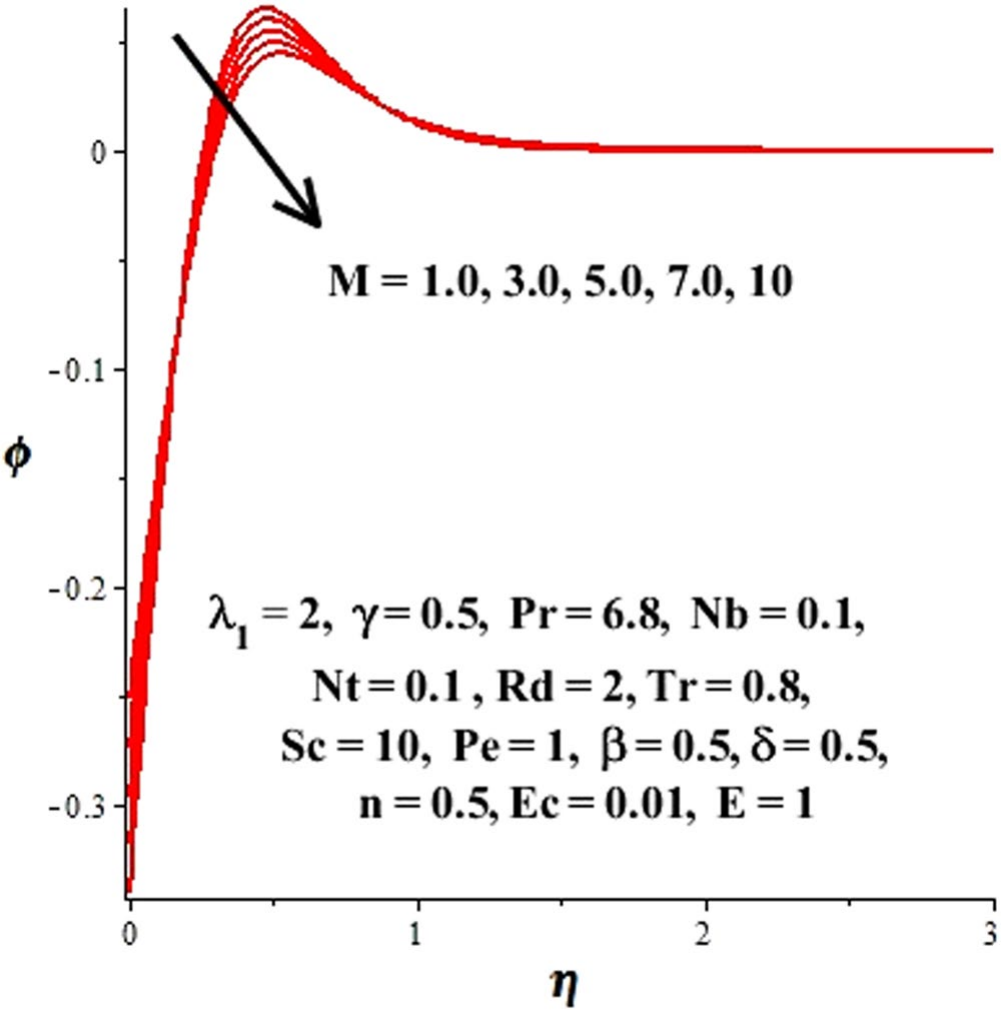
Effect of magnetic field on concentration field.

**Figure 10:**
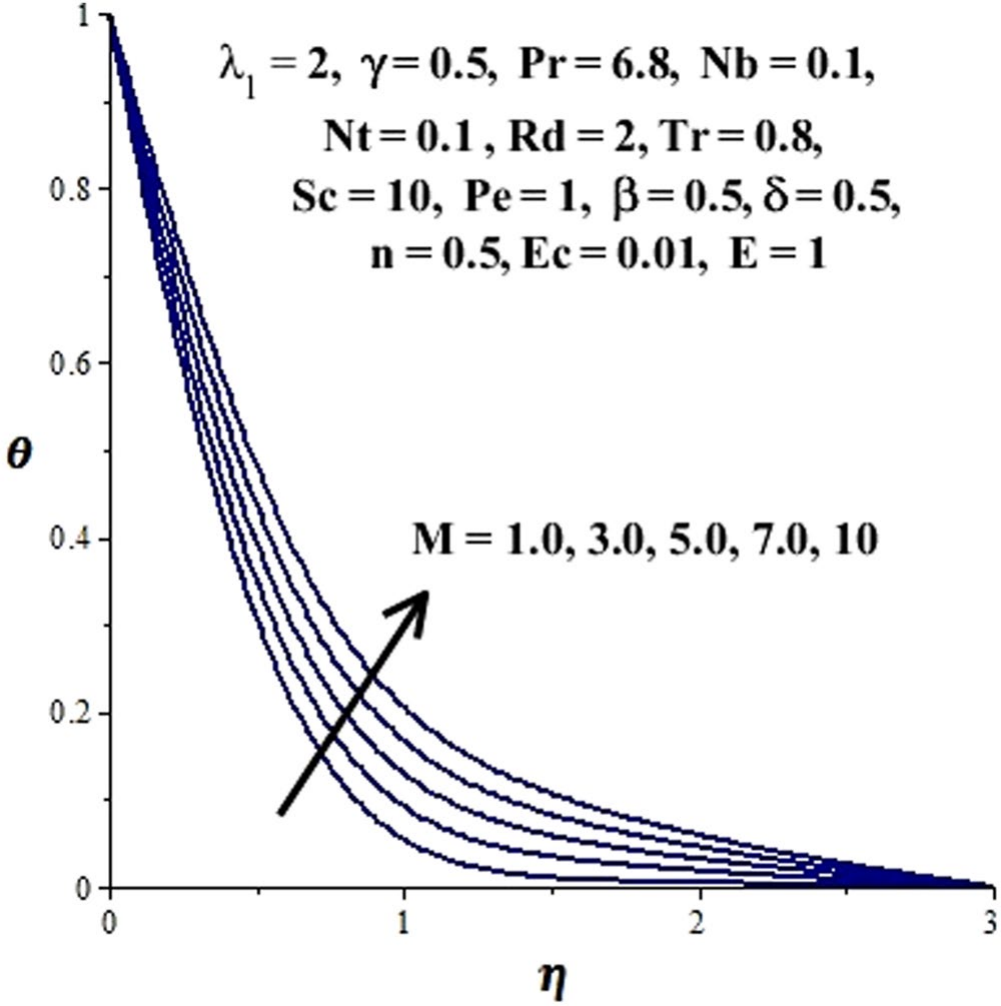
Effect of magnetic field on temperature field.

**Figure 11:**
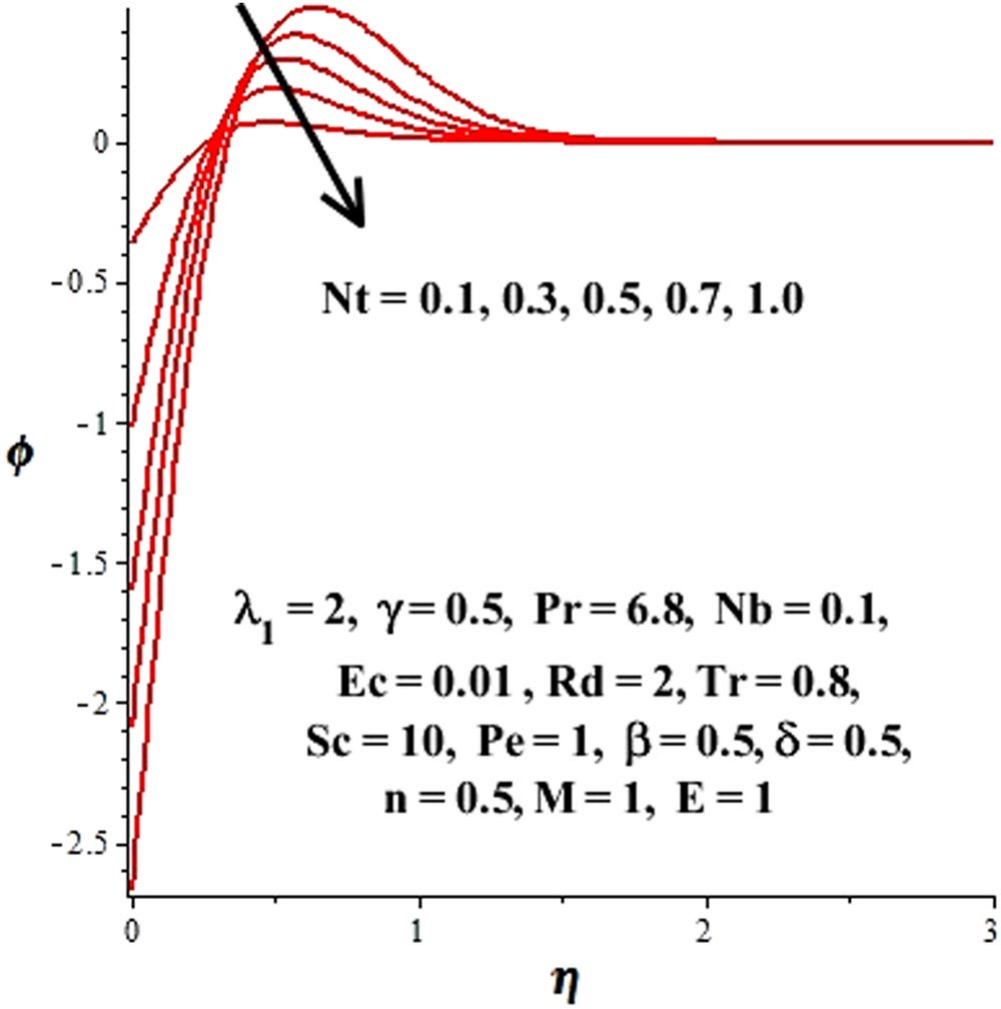
Impact of Nt on concentration distribution.

**Figure 12:**
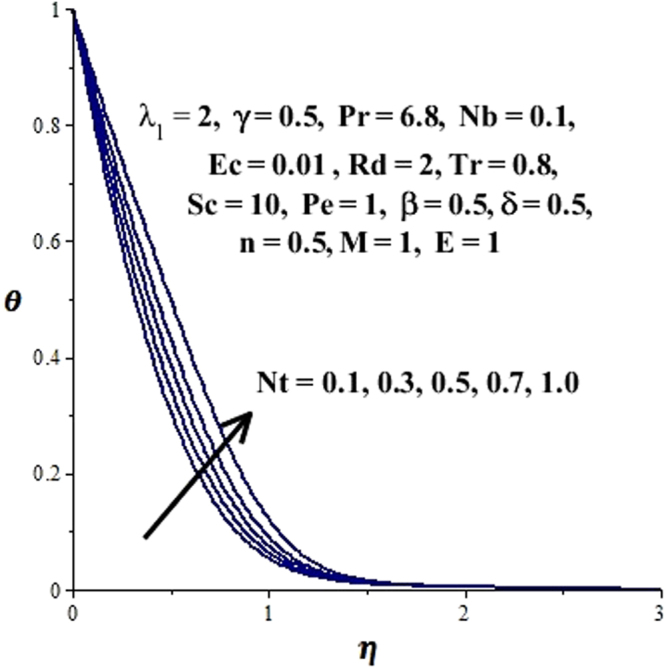
Impact of Nt on temperature field.

**Figure 13:**
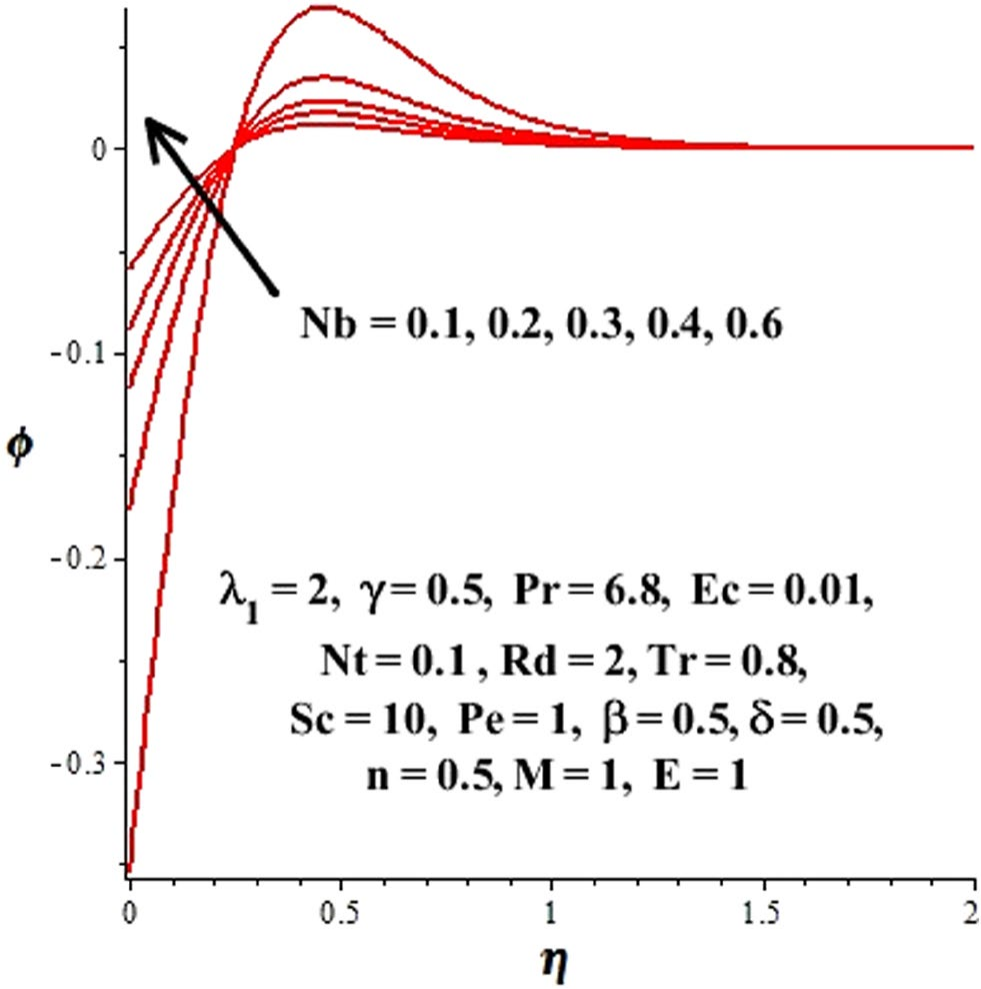
Effect of Nb on ϕ(η).

**Figure 14:**
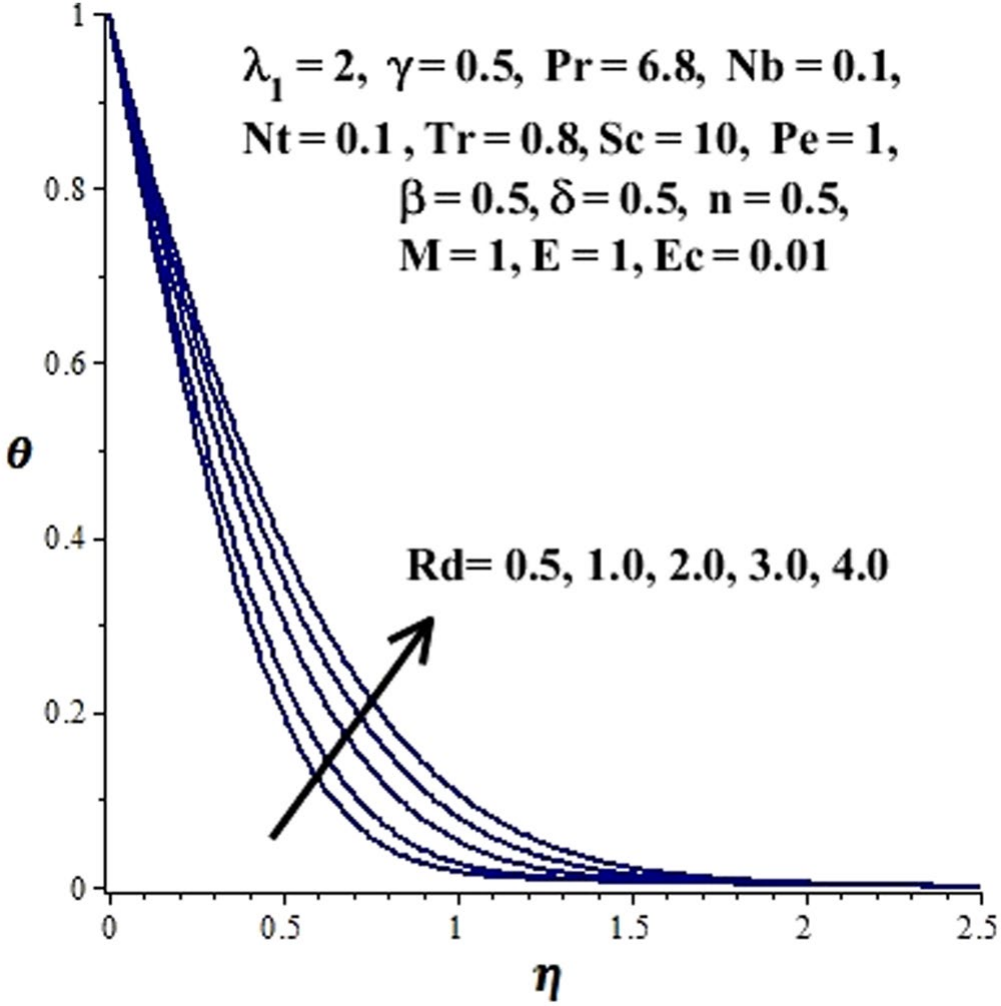
Effect of *Rd* on *θ*(η).

**Figure 15:**
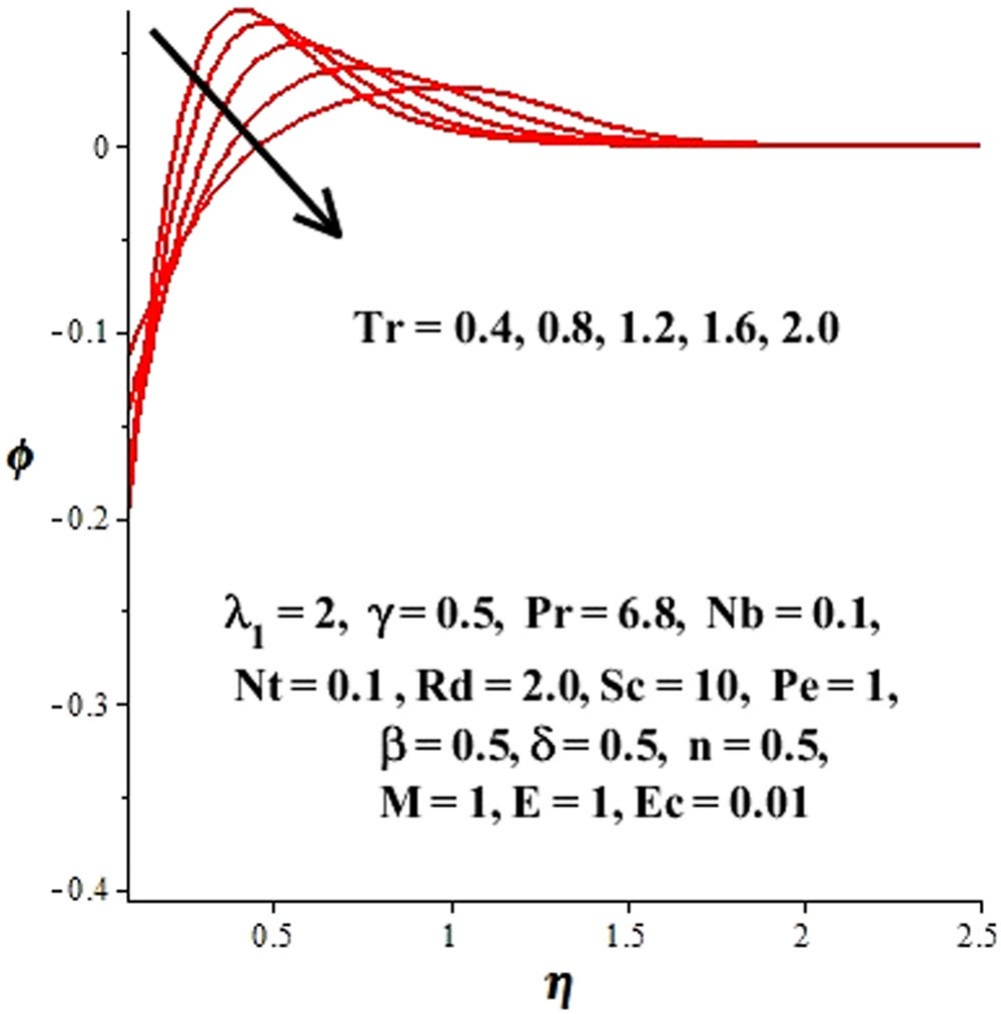
Influence of *Tr* on concentration field.

**Figure 16:**
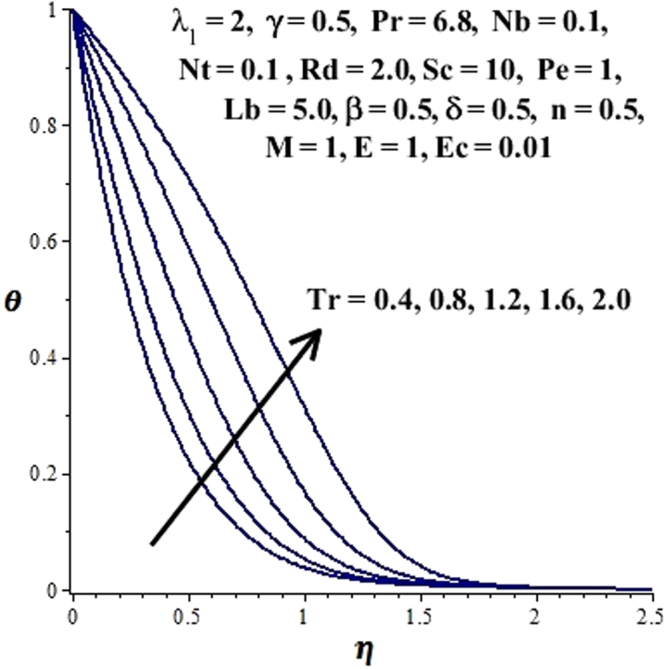
Influence of Tr on temperature profile.

**Figure 17:**
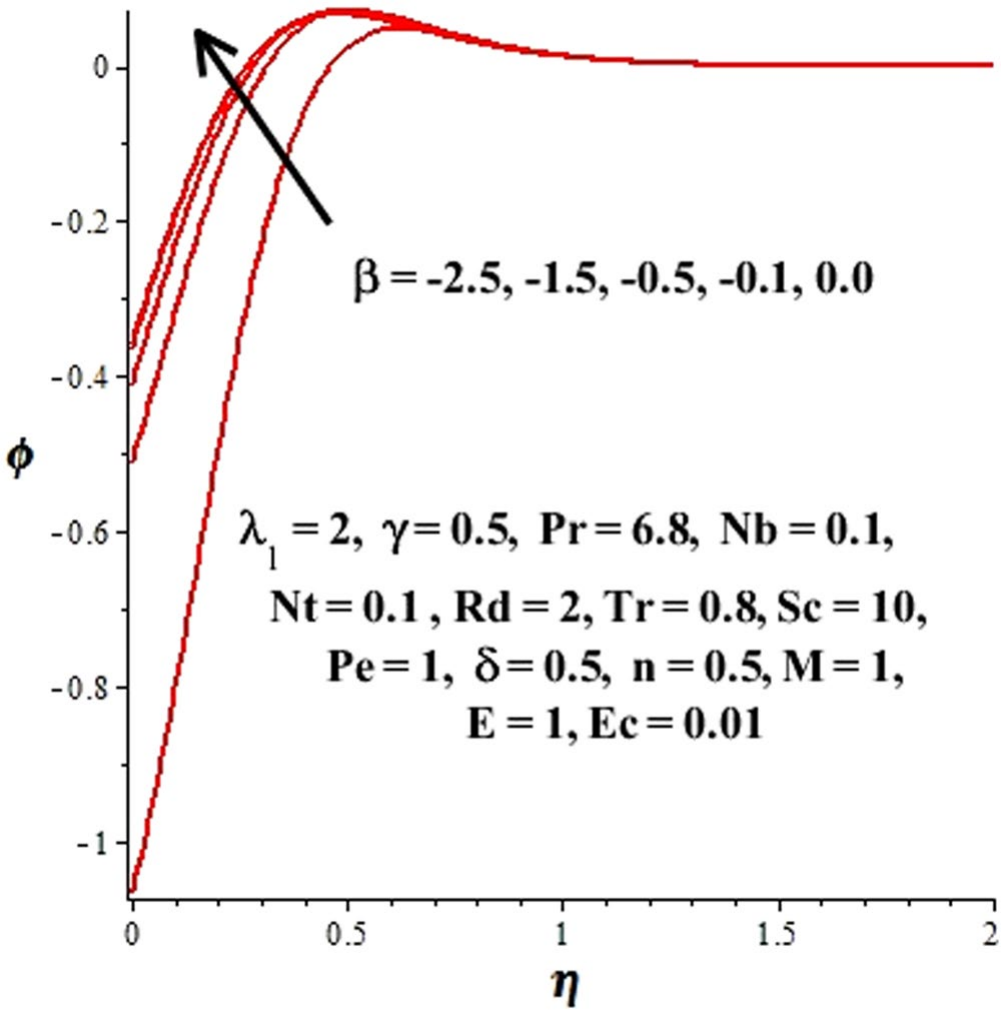
Variations of concentration distribution with respect to β ≤ 0.

**Figure 18:**
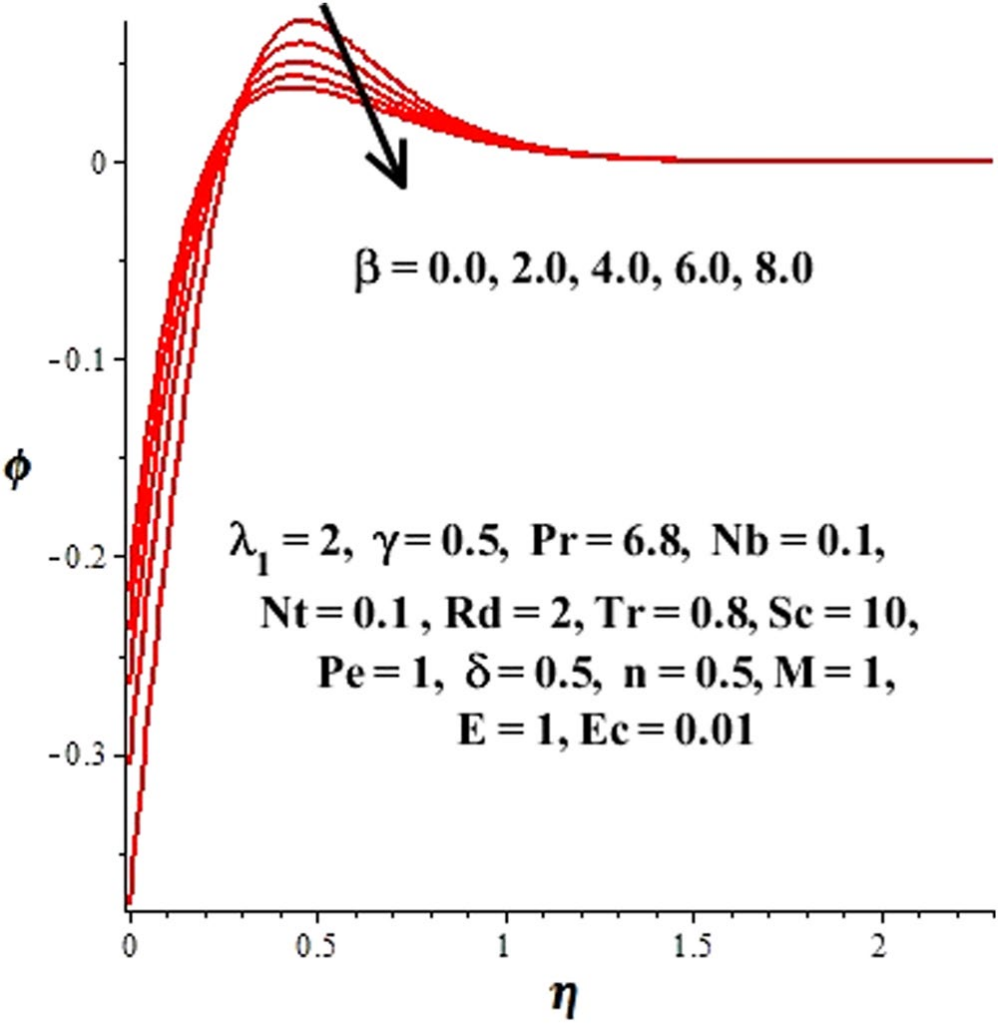
Variations of concentration distribution with respect to *β* > 0.

**Figure 19:**
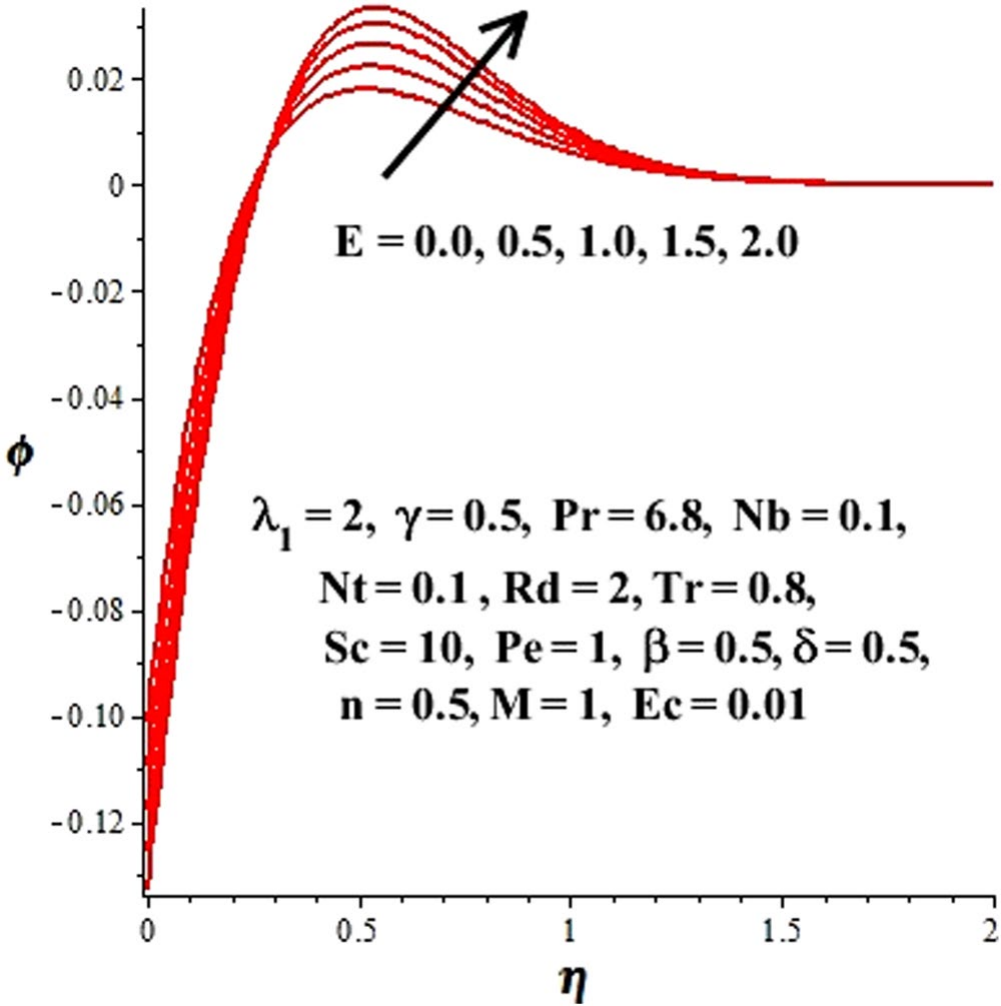
Change in ϕ(η) due to E.

**Figure 20:**
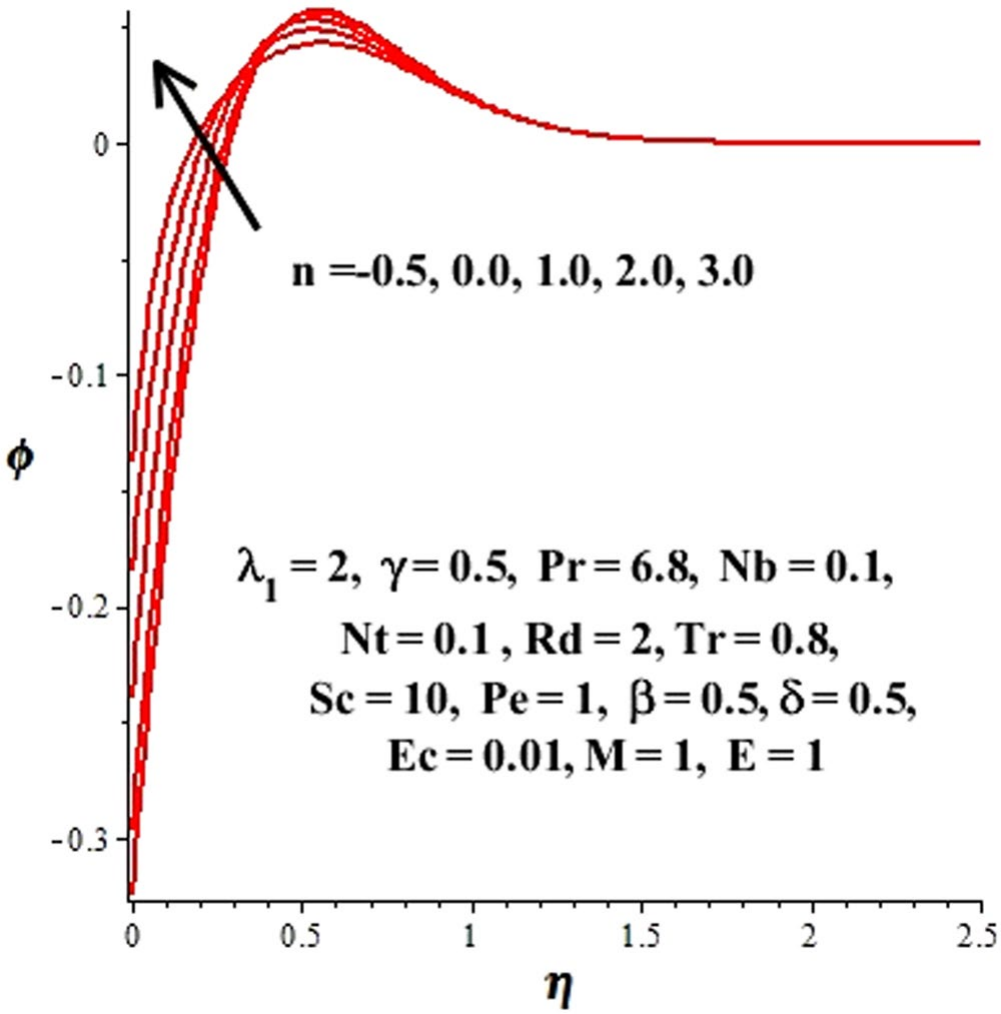
Change in ϕ(η) due to *n*.

**Figure 21:**
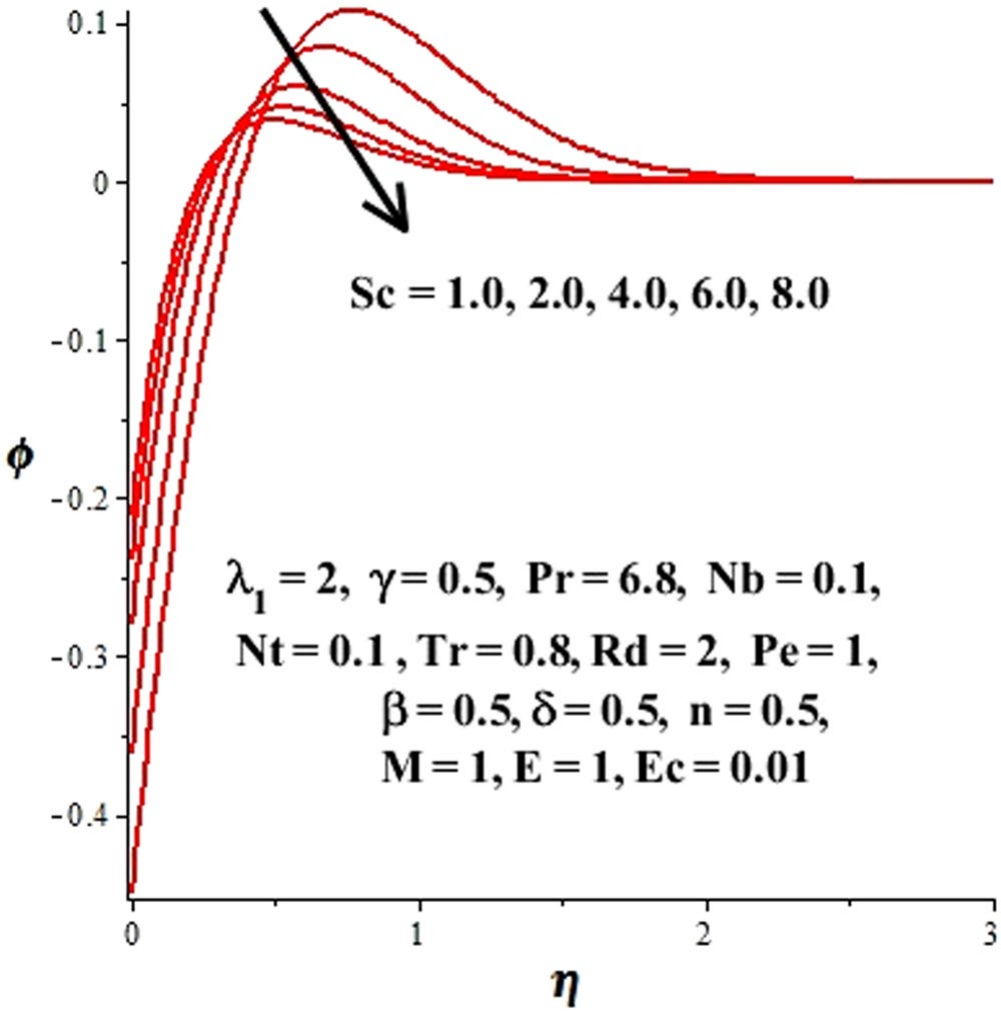
Impact of *Sc* on concentration profile.

**Figure 22:**
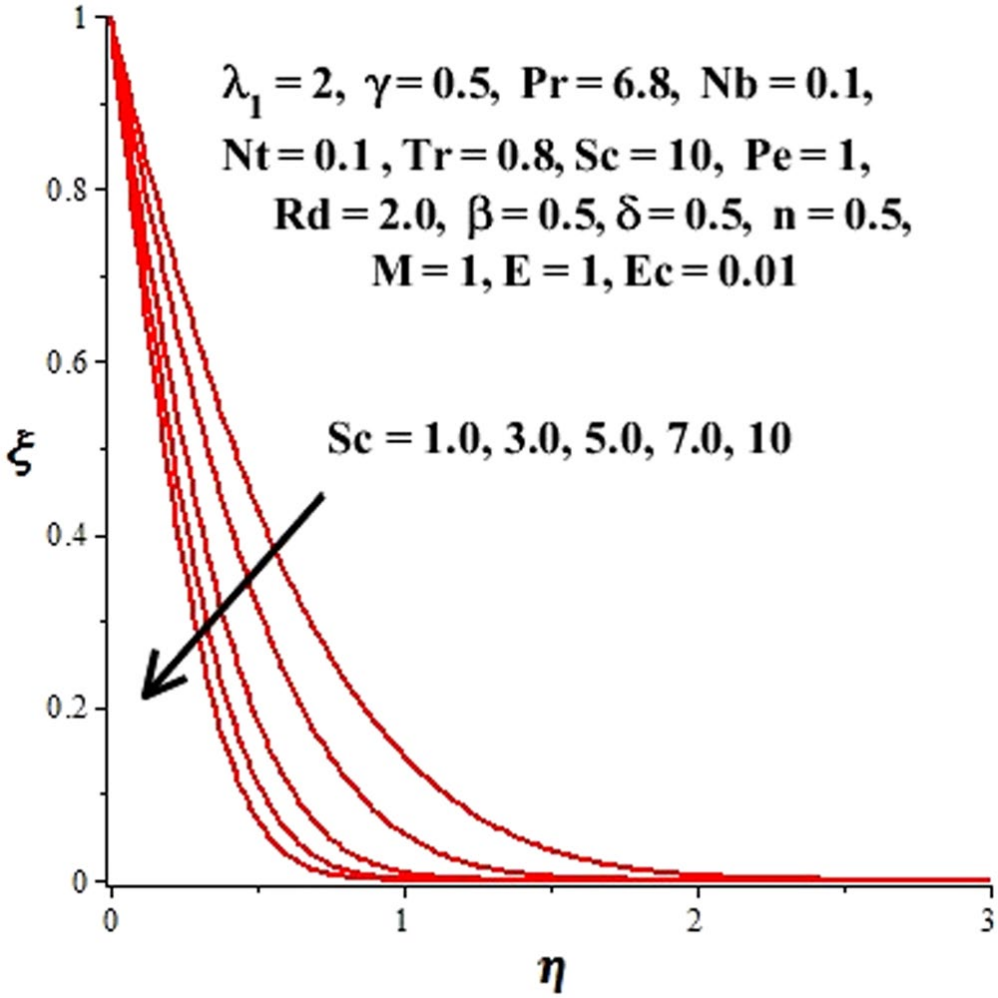
Impact of *Sc* on density of microorganisms.

**Figure 23:**
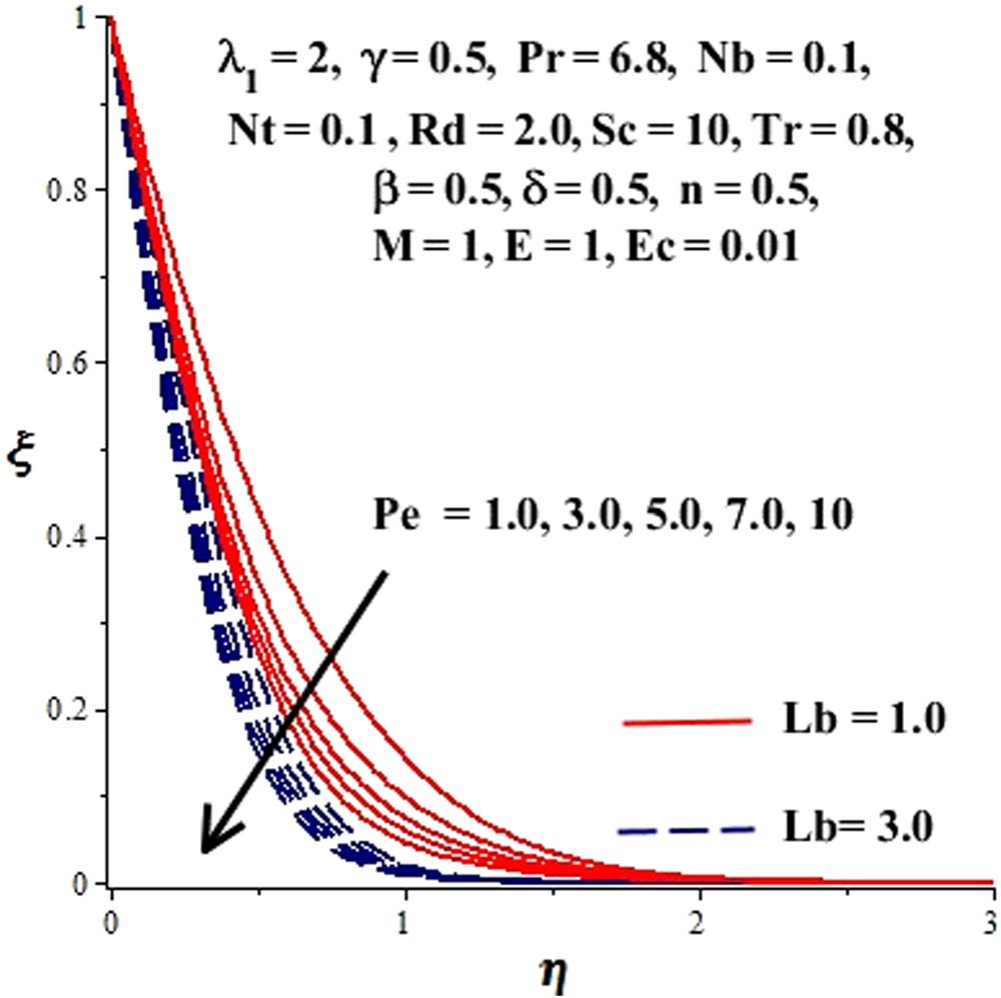
Influence of *Lb* on ξ(*η*).

**Figure 24:**
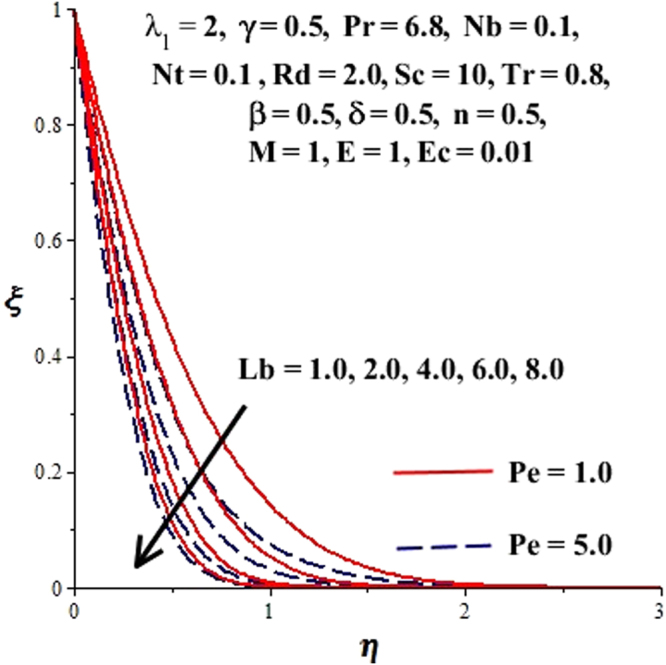
Influence of *Pe* on ξ(*η*).

In this section, we have discussed the influence of numerous physical parameters on fluid motion, heat transfer rate, concentration of the nanoparticles and density of the microorganisms. The impact is presented graphically in Figs 2 to 35. Figures (2–6) show impact of the slip factor on velocities along *x-* and *y-* axis, velocities due to lateral motion of plate, temperature profile, concentration distribution and density of microorganisms. From Fig. 2 it is seen that *f′* and *g′* enhance as the slip factor increases, this is because of decrease in shear stress due to friction. The lateral motion does not affect the velocities *f′* and *g′*, they are controlled only by stagnation flow, however, the stagnation flow affect the velocities due to lateral motion. In Fig. 3 the velocity components (due to lateral motion) *h*′, *k*′ diminish as the slip factor rises. The influence of *λ*_1_ on *θ*(*η*) is depicted in Fig. 4. It is observed that temperature field reduces for escalating values of the slip factor. This is due to reduction of the resistive forces as *λ*_1_ evolves, which increases heat transfer rate in the boundary layer. Figure 5 reveals the impact of *λ*_1_ on *ϕ*(*η*), it is observed that *ϕ*(*η*) is an increasing function of *λ*_1_, however, it decreases far away from the plate. Similarly, microorganism distribution depressed as slip factor evolves presented in Fig. 6. In Fig. 7, effect of Eckert number on the concentration profile is sketched. It can be seen that *ϕ*(*η*) declines near the surface and increases away from it, as *Ec* elevates. Similarly, the influence of *Ec* on *θ*(*η*) can be observed from Fig. 8. It is noticed that as *Ec* ascents because the heat energy is stored in the fluid due to friction which in turn enhances the fluid temperature as presented in Fig. 8. The impact of the magnetic parameter *M* on concentration and temperature profiles is portrayed in Figs 9 and 10. From Fig. 9 it is noted that *ϕ*(*η*) decreased in the boundary layer regime. Since, *M* involves Lorentz forces which resist the fluid motion and results in upsurge in temperature of the fluid. (See Fig. 10). The concentration and temperature fields are affected by thermophoretic forces as presented in Figs 11 and 12. In Fig. 11, *ϕ*(*η*) decrease near the surface and escalates away from it for increasing values of *Nt*. The thermophoretic forces push away nanoparticles from hot boundary toward fluid which results in more thickness of the thermal boundary layer and elevates the temperature of the fluid as plotted in Fig. 12. Figure 13 shows the behavior of concentration distribution for rising values of Brownian motion parameter. Since the increase in Brownian motion causes improvement of Brownian forces which boosts the concentration of nanoparticles at the surface hence *ϕ*(*η*) rise at the surface. The influence of radiation parameter on dimensionless temperature field is displayed in Fig. 14. It is reported in figure that *θ*(*η*)and thickness of corresponding thermal boundary layer notably ascents as *Rd* enhances. Physically, when *Rd* intensifies, it provides more heat to the fluid that causes thickness of thermal boundary layer. Figures 15 and 16 illustrate the variation of *ϕ*(*η*) and *θ*(*η*) for improving values of *Tr*. The concentration profile decreases for rising values of *Tr*. (See Fig. 15). Larger of values of temperature ratio parameter correspond to higher wall temperature, consequently, the temperature of the fluid and its respective thermal boundary layer improve as displayed in Fig. 16. The highlights of the influence of constructive and destructive chemical reaction on nanoparticle concentration are considered in Figs 17 and 18. For constructive case (*β* ≤ 0) the concentration distribution display increasing behavior (See Fig. 17), while it degrade for destructive chemical reaction (*β* > 0) as considered in Fig. 18. The effects of dimensionless activation energy and fitted rate constant are included in Figs 19 and 20. The increase in *E* causes a reduction in the term ${e}^{(\frac{-E}{1+\delta \theta })}$ which leads to the minimum reaction rate and consequently slow down the chemical reaction, thus the concentration of nanoparticles developed. (See Fig. 19). The rise in *ϕ*(*η*) caused by the increment of *n* is shown in Fig. 20. Figures 21 and 22 predict the behavior of Schmidt number on nanoparticle concentration and density of microorganisms. Since Schmidt number is the ratio of momentum diffusion to Brownian motion diffusion, so increase in *Sc* causes a decrease in Brownian motion diffusivity which leads to the reduction of nanoparticle concentration as evident in Fig. 21. Figure 22 reveals that the *ϕ*(*η*) decrease in magnitude as *Sc* ascents. Figures 23 and 24 give the impact of bioconvection Peclet number and bioconvection Lewis number on the density of microorganisms. As the Peclet number is the fraction of maximum cell swimming speed and diffusion of microorganisms. So raising values of *Pe* implies a decrease in diffusion of microorganism which results in a diminishing of the density of microorganism. (See Fig. 23). Similarly, from Fig. 24 the density of microorganism declines for escalating values of the bioconvection Lewis number *Lb*.

**Figure 25:**
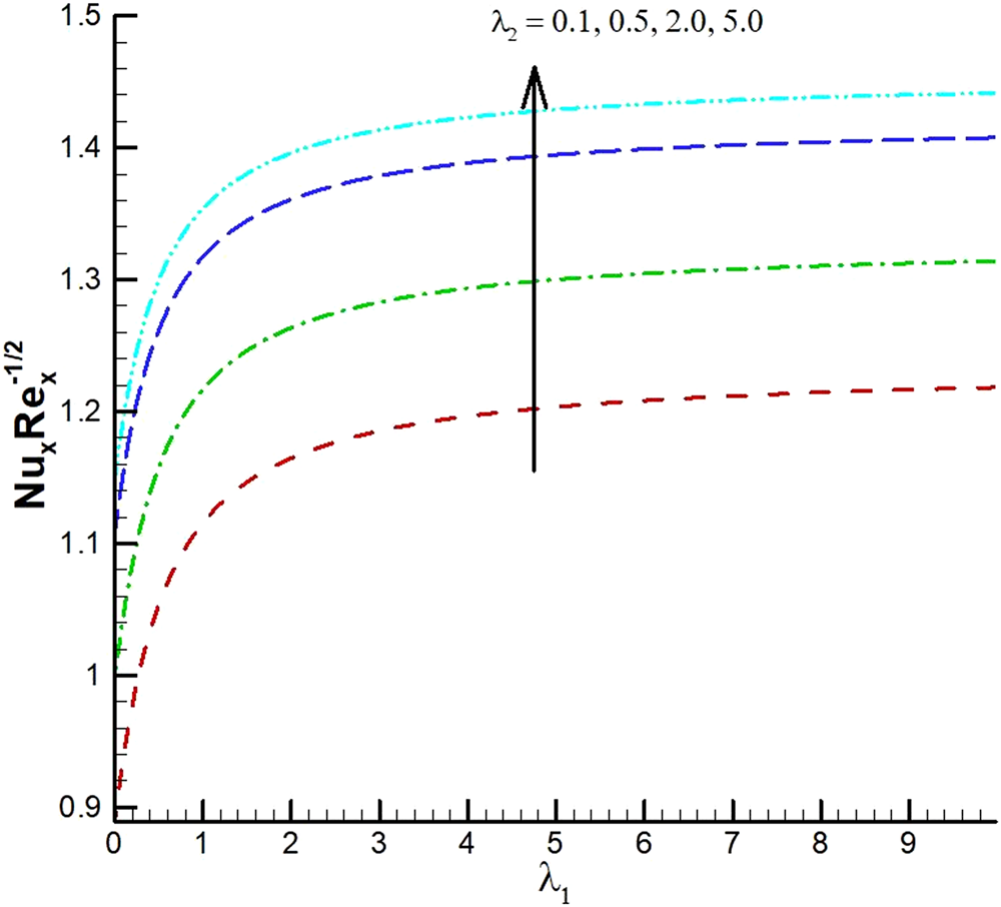
Variations of Nusselt number for various values of λ_2_ and λ_1_.

**Figure 26:**
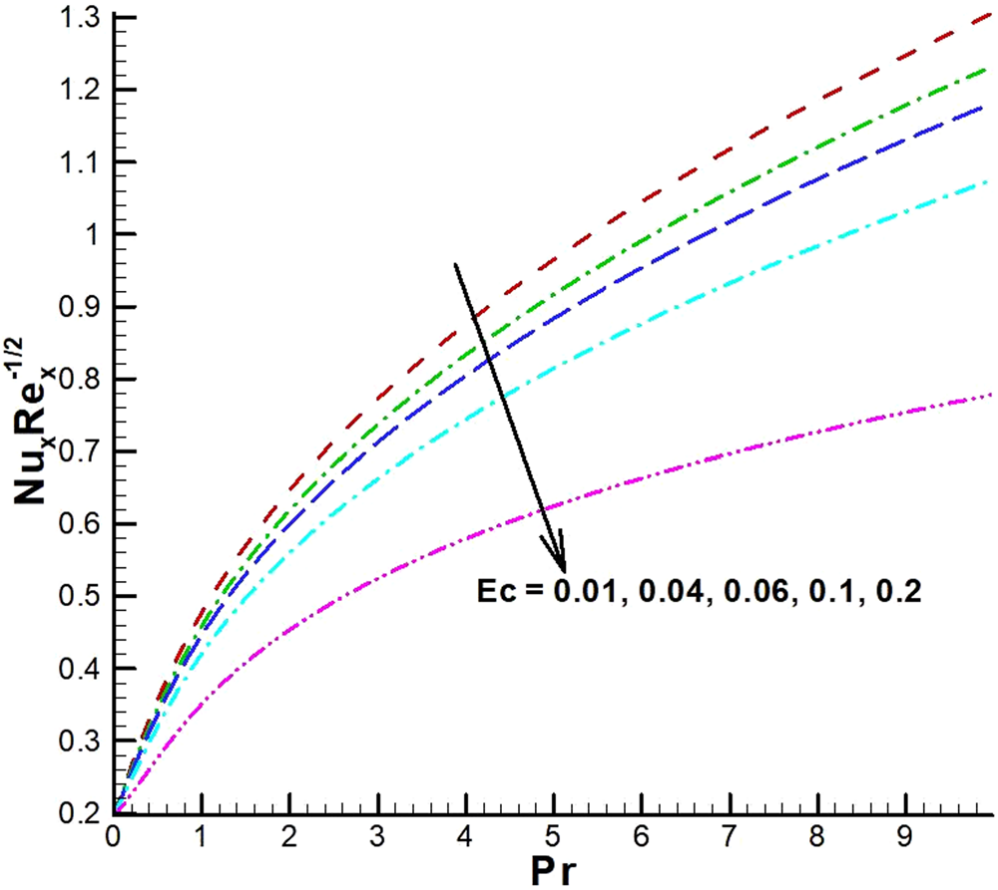
Variations of Nusselt number for various values of *Ec* and *Pr*.

**Figure 27:**
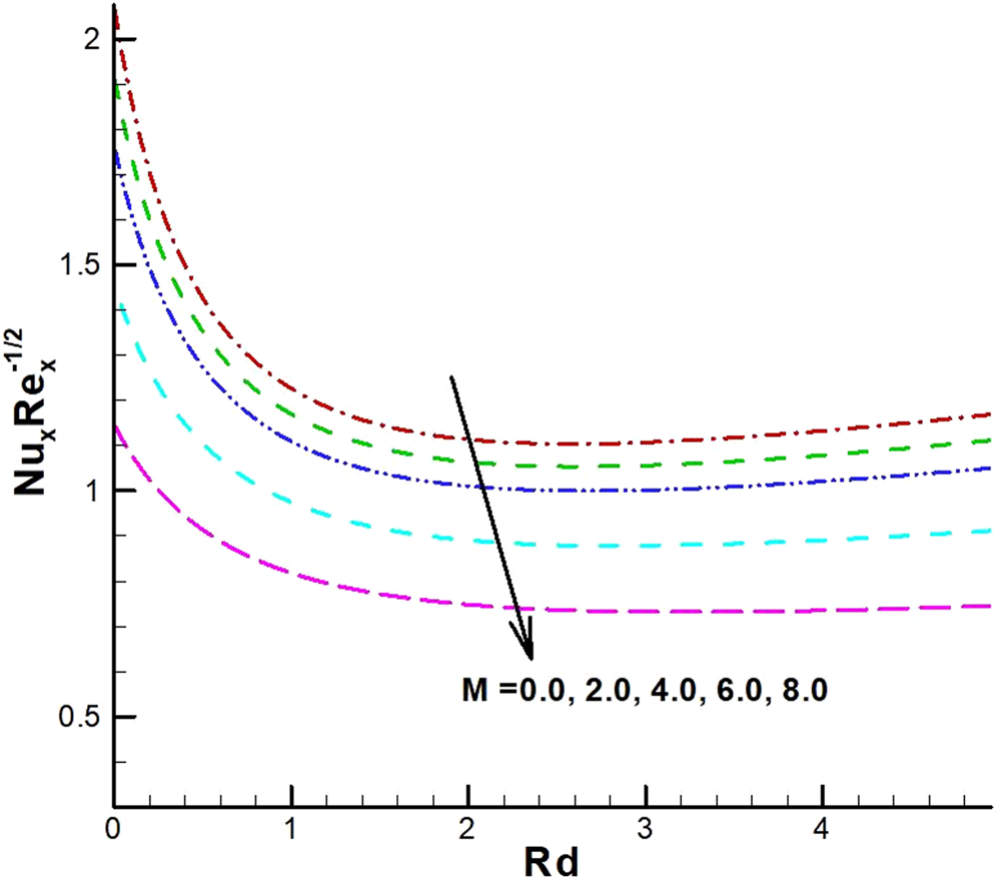
Variations of Nusselt number for various values of *M* and *Rd*.

**Figure 28:**
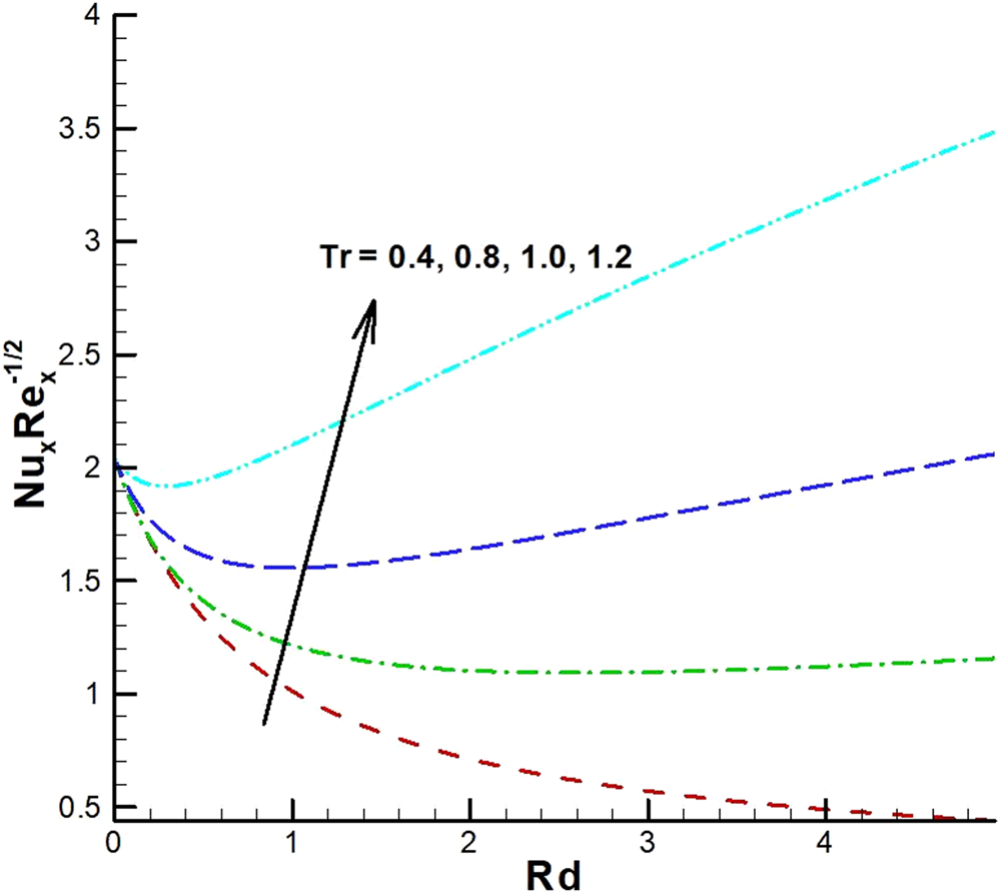
Variations of Nusselt number for various values of *Tr and Rd*.

**Figure 29:**
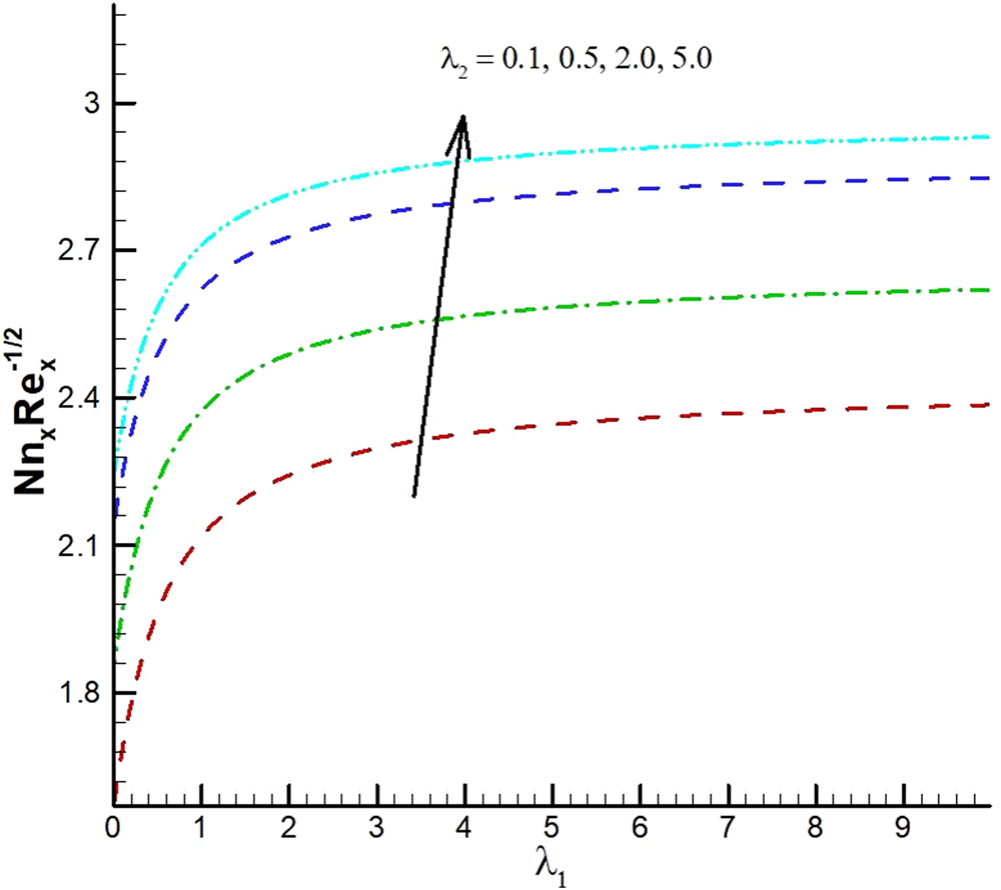
Variations of motile density number for various values of λ_2_ and λ_1_.

**Figure 30:**
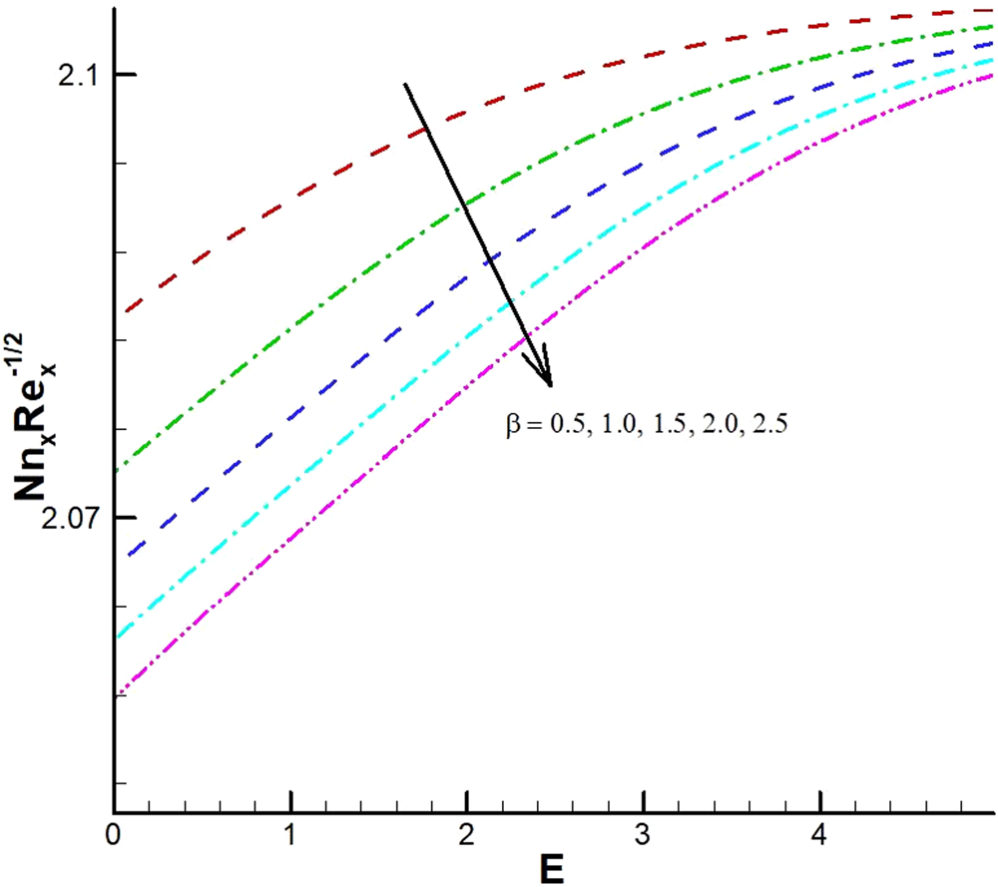
Variations of motile density number for various values of *β* and *E*.

**Figure 31:**
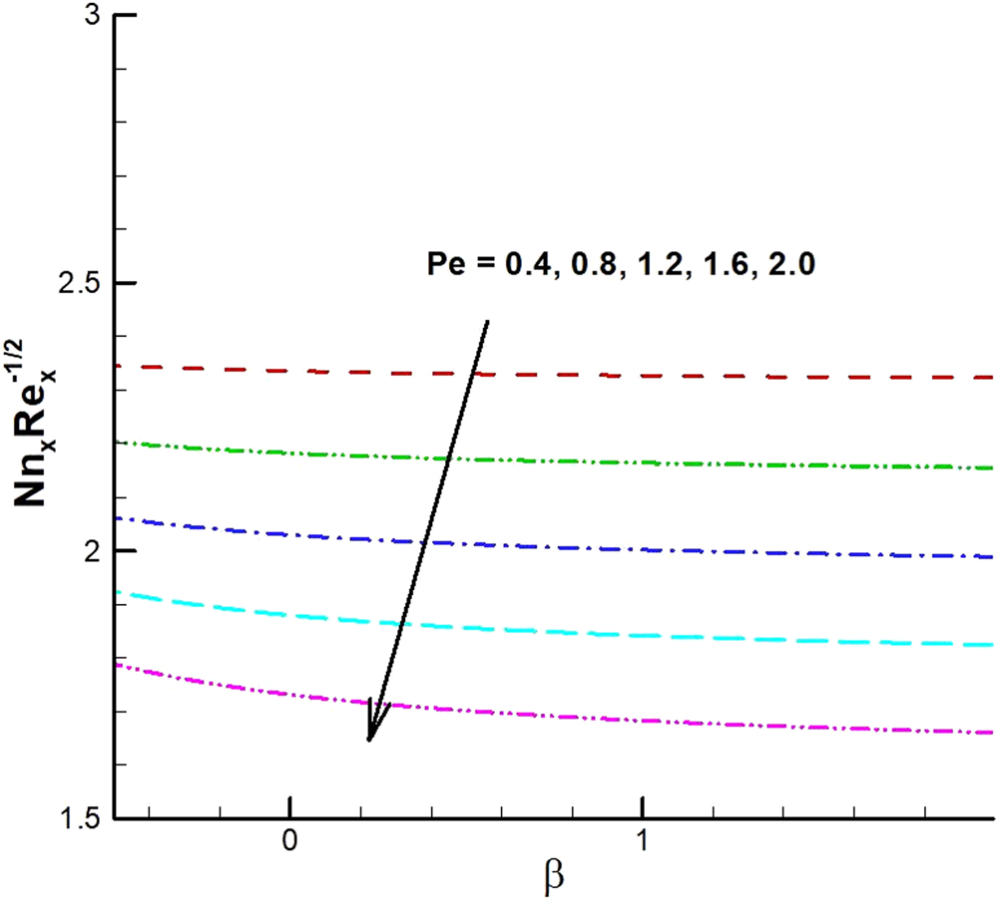
Variations of motile density number for various values of *Pe* and *β*.

**Figure 32:**
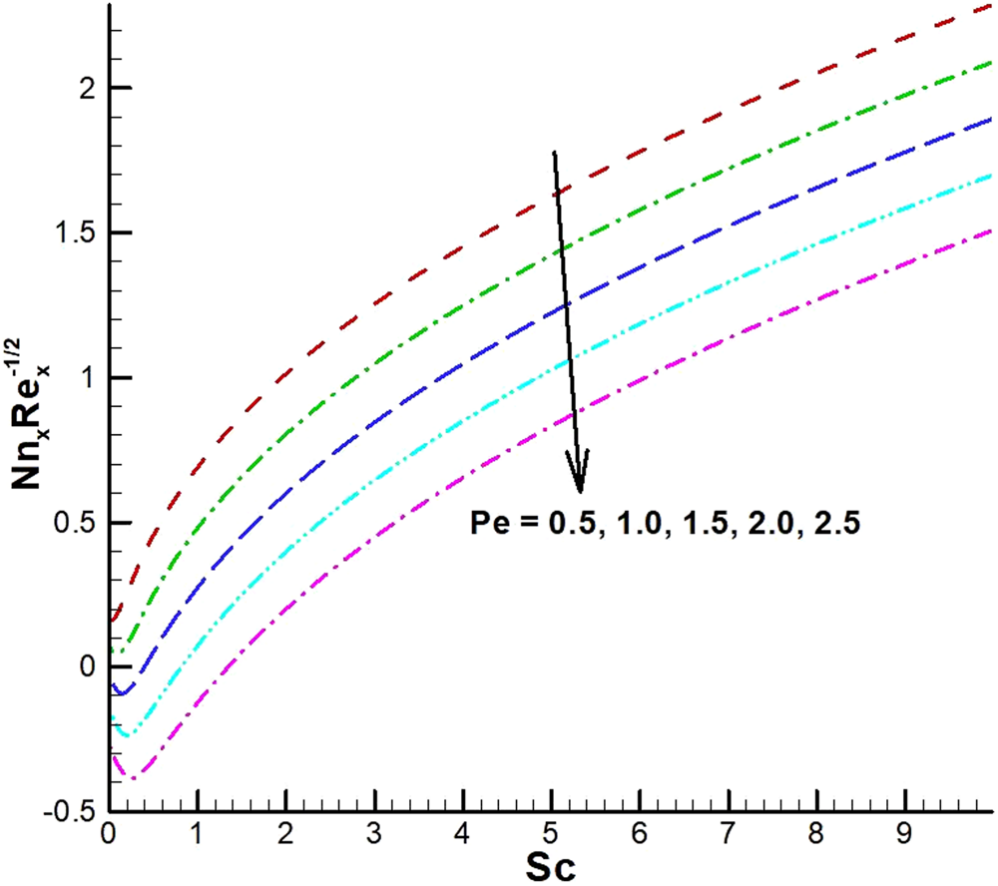
Variations of motile density number for various values of *Pe and Sc*.

**Figure 33:**
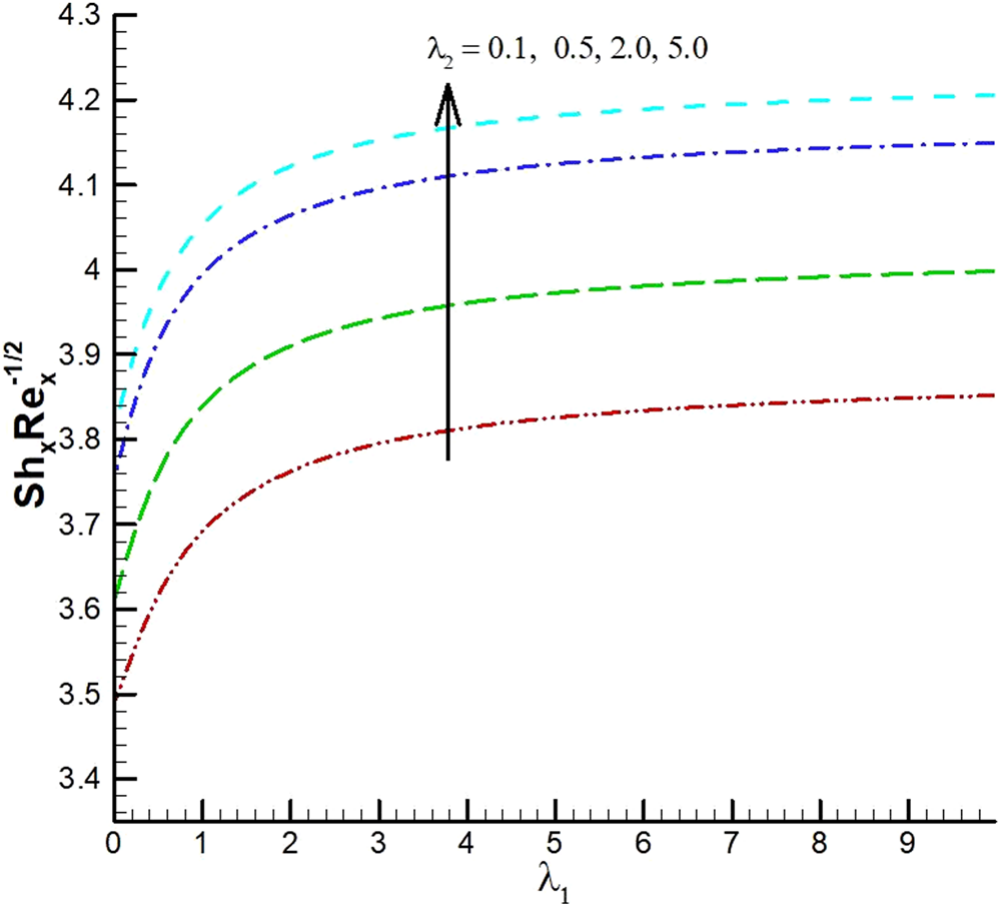
Variations of local Sherwood number for various values of λ_2_ and λ_1_.

**Figure 34:**
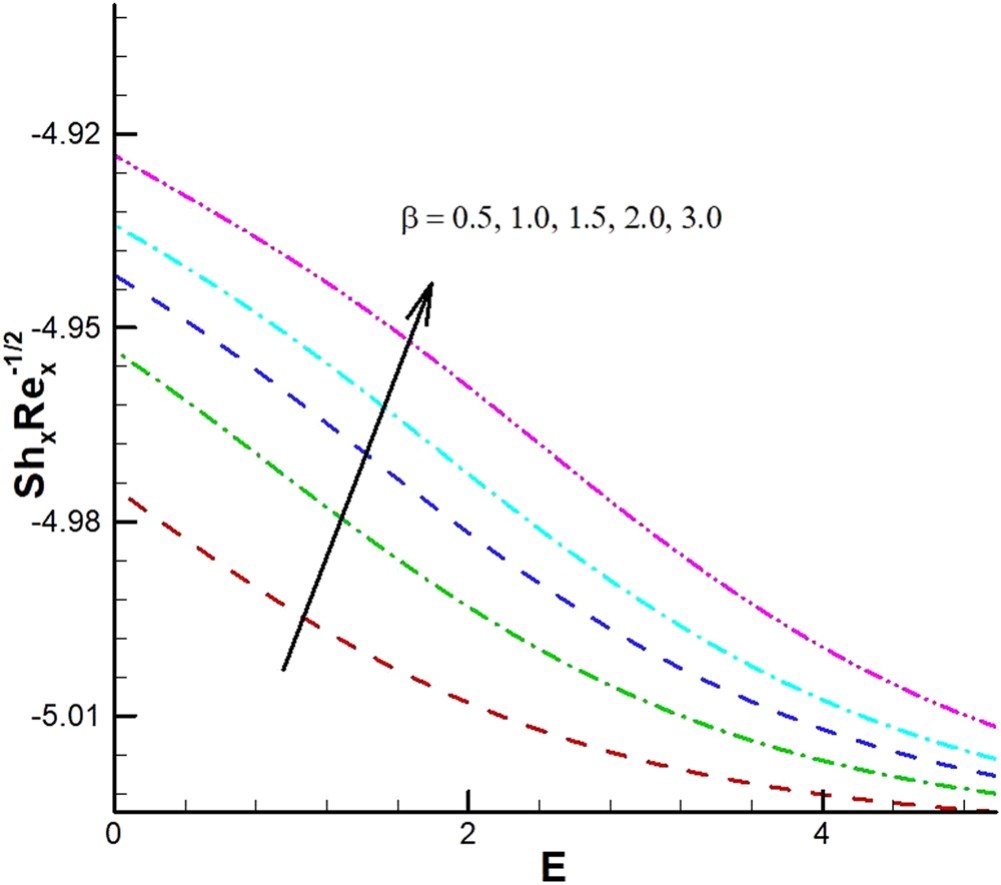
Variations of local Sherwood number for various values of *β* and *E*.

**Figure 35:**
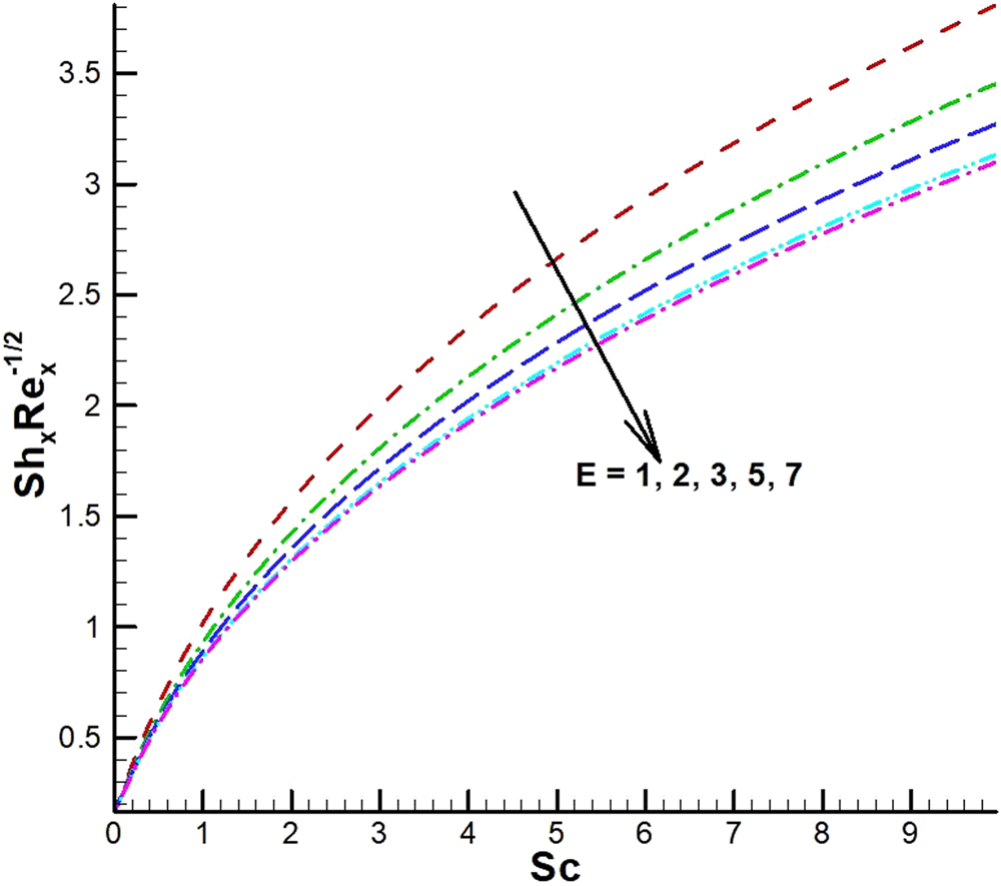
Variations of local Sherwood number for various values of *E* and *Sc*.

The behavior of reduced Nusselt number, Sherwood number and local density of microorganisms against different emerging parameters are presented in Figs 25–35. Figure 25 gives the increasing behavior of Nusselt number for rising values of slip parameter *λ*_2_ versus slip factor *λ*_1_. The effect of viscous dissipation parameter (Eckert number) and Prandtl number is presented in Fig. 26. It is observed that rate of heat transfer against the Prandtl number decreases rapidly for rising values of *Ec*. Similarly, the magnitude of heat transfer rate against radiation parameter for various values of *M* and *Tr* is displayed in Figs 27 and 28. From Fig. 27 the Nusselt number against *Rd* is a decreasing function of the magnetic parameter, whereas it gives rising values for temperature ratio parameter. (See Fig. 28). The influence of different parameters on motile microorganism number is portrayed in Figs 29–32. From Fig. 29 the incremented behavior of $-\xi \text{'}(0)$ can be observed for increasing *λ*_2_ versus *λ*_1_. Microorganism flux density falls in behavior for rising in chemical reaction rate constant. This effect against dimensionless activation energy is presented in Fig. 30. Inspections of Figs 31 and 32 predict that motile density of microorganism exhibits decreasing magnitude for bioconvection Peclet number versus chemical reaction parameter and Schmidt number. Figures 33–35 describe the variation of Sherwood number for various values of different parameters. It can be observed from Figs 33 and 34 that Sherwood number increases for enhancing values of slip parameter *λ*_2_ and chemical reaction rate constant. The impact of dimensionless activation energy and Schmidt number is presented in Fig. 34. From Fig. 35 it can be noted that Sherwood number is a decreasing function of *E*.

## Conclusion

Through this analysis we have explored three-dimensional stagnation flow of the nanofluid containing gyrotactic microorganism on a moving surface with anisotropic slip. Furthermore, influence of viscous dissipation and Joule heating, nonlinear thermal radiation, binary chemical reaction and activation energy effects are also considered. A numerical method is adopted to tackle the non-linear system of ordinary differential equations. Following are the leading features of the present exploration:

- Heat transfer rate diminishes for improving values of the Eckert number and magnetic parameter.
- There is an enhancement in local Nusselt number for escalating temperature ratio parameter versus radiation parameter.
- The slip parameter has an increasing behavior on density of motile microorganism, while it shows inverse trend for rising values of the reaction rate constant.
- The decreasing magnitude of density of microorganisms is observed for bioconvection Peclet number against chemical reaction rate constant and Schmidt number.
- The slip effect and chemical reaction parameter has increasing behavior on local Sherwood number.
- It is noticed that the local density number of microorganisms decline for dimensionless activation energy against Schmidt number.
